# Alcohol and Liver Clock Disruption Increase Small Droplet Macrosteatosis, Alter Lipid Metabolism and Clock Gene mRNA Rhythms, and Remodel the Triglyceride Lipidome in Mouse Liver

**DOI:** 10.3389/fphys.2020.01048

**Published:** 2020-09-07

**Authors:** Jennifer A. Valcin, Uduak S. Udoh, Telisha M. Swain, Kelly K. Andringa, Chirag R. Patel, Sameer Al Diffalha, Paul R. S. Baker, Karen L. Gamble, Shannon M. Bailey

**Affiliations:** ^1^Division of Molecular and Cellular Pathology, Department of Pathology, University of Alabama at Birmingham, Birmingham, AL, United States; ^2^Division of Nephrology, Department of Medicine, University of Alabama at Birmingham, Birmingham, AL, United States; ^3^Division of Anatomic Pathology, Department of Pathology, University of Alabama at Birmingham, Birmingham, AL, United States; ^4^Avanti Polar Lipids, Inc., Alabaster, AL, United States; ^5^Department of Psychiatry and Behavioral Neurobiology, University of Alabama at Birmingham, Birmingham, AL, United States

**Keywords:** liver, alcohol, circadian clock, BMAL1, steatosis, triglyceride, fatty acid, lipidomics

## Abstract

Heavy alcohol drinking dysregulates lipid metabolism, promoting hepatic steatosis – the first stage of alcohol-related liver disease (ALD). The molecular circadian clock plays a major role in synchronizing daily rhythms in behavior and metabolism and clock disruption can cause pathology, including liver disease. Previous studies indicate that alcohol consumption alters liver clock function, but the impact alcohol or clock disruption, or both have on the temporal control of hepatic lipid metabolism and injury remains unclear. Here, we undertook studies to determine whether genetic disruption of the liver clock exacerbates alterations in lipid metabolism and worsens steatosis in alcohol-fed mice. To address this question, male liver-specific *Bmal1* knockout (LKO) and flox/flox (Fl/Fl) control mice were fed a control or alcohol-containing diet for 5 weeks. Alcohol significantly dampened diurnal rhythms of mRNA levels in clock genes *Bmal1* and *Dbp*, phase advanced *Nr1d1*/REV-ERBα, and induced arrhythmicity in *Clock*, *Noct*, and *Nfil3*/E4BP4, with further disruption in livers of LKO mice. Alcohol-fed LKO mice exhibited higher plasma triglyceride (TG) and different time-of-day patterns of hepatic TG and macrosteatosis, with elevated levels of small droplet macrosteatosis compared to alcohol-fed Fl/Fl mice. Diurnal rhythms in mRNA levels of lipid metabolism transcription factors (*Srebf1*, *Nr1h2*, and *Ppara*) were significantly altered by alcohol and clock disruption. Alcohol and/or clock disruption significantly altered diurnal rhythms in mRNA levels of fatty acid (FA) synthesis and oxidation (*Acaca/b*, *Mlycd*, *Cpt1a*, *Fasn*, *Elovl5/6*, and *Fads1/2*), TG turnover (*Gpat1*, *Agpat1/2*, *Lpin1/2*, *Dgat2*, and *Pnpla2/3*), and lipid droplet (*Plin2/5*, *Lipe*, *Mgll*, and *Abdh5*) genes, along with protein abundances of p-ACC, MCD, and FASN. Lipidomics analyses showed that alcohol, clock disruption, or both significantly altered FA saturation and remodeled the FA composition of the hepatic TG pool, with higher percentages of several long and very long chain FA in livers of alcohol-fed LKO mice. In conclusion, these results show that the liver clock is important for maintaining temporal control of hepatic lipid metabolism and that disrupting the liver clock exacerbates alcohol-related hepatic steatosis.

## Introduction

Alcohol use is a top 10 cause of preventable death in the United States, with alcohol-related liver disease (ALD) the number one cause of death from excessive alcohol consumption (Minino et al., 2006; Danaei et al., 2009). Hepatic steatosis, the accumulation of triglyceride (TG)-rich lipid droplets in the cytosol of hepatocytes, is the first response that occurs in the liver following heavy alcohol drinking. Alcohol metabolism increases the hepatic NADH/NAD^+^ ratio (Bailey and Cunningham, 1998), which inhibits mitochondrial fatty acid oxidation (FAO; Cederbaum et al., 1975; Salaspuro et al., 1981) and increases *de novo* lipogenesis (You and Arteel, 2019). Moreover, alcohol impacts lipid metabolism at the transcriptional level by affecting SREBP-1c (You et al., 2002), PPARα and γ (Abdelmegeed et al., 2011; Zhang et al., 2016b, 2018), and LXR-mediated pathways (Sengupta et al., 2018). Alcohol also impairs lipid droplet turnover and lipophagy, which contribute to the development of hepatic steatosis (Orlicky et al., 2011; Carr et al., 2013; Yang et al., 2019). Steatosis is recognized as a crucial first step in the initiation and progression of ALD by sensitizing hepatocytes to additional metabolic stressors, leading to cell death and disease (Spach et al., 1991; Jou et al., 2008; Zelickson et al., 2011).

Steatosis is divided into two types – macrosteatosis and microsteatosis. Macrosteatosis is characterized by the presence of large lipid droplets, whereas microsteatosis is characterized by the presence of many tiny cytosolic lipid droplets giving hepatocytes a foamy-like appearance (Brunt, 2007; Yeh and Brunt, 2014). Macrosteatosis occurs with acute and chronic alcohol consumption in humans and experimental animal models (Sakhuja, 2014). In contrast, microsteatosis is more rare and observed during severe clinical situations, such as acute fatty liver of pregnancy (Brady, 2020), Reyes syndrome in children (Glasgow and Middleton, 2001), and drug-induced hepatotoxicity (Begriche et al., 2011) in response to significant mitochondrial damage and impaired FAO (Fromenty and Pessayre, 1995; Fromenty and Pessayre, 1997). Microsteatosis is also found in ALD patients, but is uncommon (Theise, 2013). Recent guidelines suggest that macrosteatosis be classified as a mixture of large and small lipid droplets. In small droplet macrosteatosis, smaller droplets of varying sizes are present in the cytosol with the nucleus remaining in the center of hepatocytes. On the other hand, in large droplet macrosteatosis, the nucleus is displaced to one side of the cell by a large unilocular lipid droplet (Mostafa et al., 2020). As highlighted by Mostafa et al. (2020), this classification is highly important as small droplet macrosteatosis is commonly mistaken for microsteatosis by researchers. Importantly, the molecular events underpinning these different steatosis phenotypes and their pathological significance are poorly understood. One possible reason for this is that there are still large gaps in knowledge regarding lipid metabolism, including an incomplete understanding regarding how alcohol affects diurnal regulation of lipid metabolism, including effects on the molecular circadian clock, a key regulator of metabolism (Filiano et al., 2013; Zhou et al., 2014; Zhang et al., 2018).

Biological circadian rhythms are regulated by interactions of multiple inputs from sources that are external (light/dark and feeding/fasting cycles) and internal (molecular clock). The clock is a hierarchal system comprised of molecular oscillators found throughout the body that synchronize daily rhythms in behavior, metabolism, and physiology to maintain normal organ function and overall whole body health (Bailey et al., 2014; Takahashi, 2017; Koronowski et al., 2019). Circadian rhythms are generated at the molecular level by a system of transcriptional-translational feedback loops. In the first loop, the transcription factors brain and muscle ARNT-like 1 (BMAL1) and circadian locomotor output cycles kaput (CLOCK) hetero-dimerize and bind to gene promoter E-box motifs to activate transcription of *Period 1*, *2*, or *3* (*Per1–3*) and *Cryptochrome 1* or *2* (*Cry1–2*) genes, whose protein products (PER1–3, CRY1–2) feedback to inhibit BMAL1-CLOCK and their own transcription (Ko and Takahashi, 2006). A second feedback loop is formed by the retinoid-related orphan receptors (ROR) and nuclear receptor subfamily 1 group D members 1 and 2 (NR1D1 and 2; a.k.a., REV-ERBα and β), which activate and inhibit *Bmal1* transcription, respectively (Preitner et al., 2002; Sato et al., 2004). BMAL1-CLOCK also drives diurnal rhythms in mRNA levels of numerous non-clock and metabolic genes in the liver, e.g., approximately 10% of transcripts oscillate in a BMAL1-dependent manner in livers of normal wild-type mice (Koronowski et al., 2019).

Disruption in circadian clocks and their metabolic and functional outputs lead to pathology. For example, whole-body *Bmal1* knockout mice and *Clock^Δ19/Δ19^* mutant mice have severe dyslipidemia (Turek et al., 2005; Shimba et al., 2011). Altered lipid and cholesterol metabolism also occur in mice with liver-specific deletion of *Bmal1* and *Nr1d1*/REV-ERBα (Bugge et al., 2012; Cho et al., 2012; Zhang et al., 2014). Both genetic (*Clock^Δ19/Δ19^* mice) and environmental (chronic phase shifts of the light/dark cycle) circadian disruption exacerbate alcohol-related pathologies, including increased intestinal permeability and hepatic inflammation (Summa et al., 2013; Voigt et al., 2016). Similarly, we have reported that liver clock disruption (by *Bmal1* deletion in hepatocytes) significantly disrupts 24 h rhythms in mRNA levels of glycogen and glucose metabolism genes and glycogen content in livers of alcohol-fed mice (Udoh et al., 2017). Based on this, we hypothesized that genetic disruption of the liver clock would exacerbate alcohol-related impairments in lipid metabolism and worsen steatosis in the liver. To test this hypothesis, we determined clock and lipid metabolism mRNA levels, hepatic and plasma TG levels, liver injury, and the fatty acid (FA) profile of the hepatic TG pool, i.e., TG lipidome, in mice with liver-specific knockout of *Bmal1* fed a control or alcohol-containing diet.

## Materials and Methods

### Chronic Alcohol Feeding Procedure

Eight to ten week old male liver-specific *Bmal1* knockout mice (LKO mice: *Alb^Cre+/−^*; *Bmal1^Fl/Fl^*) and control littermates (Fl/Fl mice: *Alb^Cre−/−^*; *Bmal1^Fl/Fl^*) on a C57BL/6J background were weight-matched into pairs and then assigned into a control or alcohol (ethanol) diet feeding group as described in our previous study (Udoh et al., 2017). Mice were pair-fed iso-caloric (1 kcal/ml) liquid diets (control diet: F1259SP and ethanol diet: F1258SP) from Bio-Serv (Frenchtown, NJ; Filiano et al., 2013). Mice in the alcohol-fed group were acclimated to the diet by gradually increasing the concentration of alcohol in the diet from 0 to 3% (w/v) over a 1 week period and maintained at this level for 4 weeks (Udoh et al., 2017). This concentration of alcohol (3%, w/v) in the diet is equivalent to 22% total daily caloric intake. This alcohol-feeding protocol is widely used and well accepted as a model of the earliest stage of ALD – hepatic steatosis (Lieber et al., 1989; Brandon-Warner et al., 2012). Mice were single-housed in standard cages with bedding and kept in a temperature-, humidity-, and light-controlled room with a 12-h light:12-h dark (12-h L:D) schedule. The amount of liquid diet consumed by each mouse was measured every 24 h with diets replaced daily between zeitgeber time (ZT) 10–12 (ZT 0 = beginning of lights on/inactive period and ZT 12 = beginning of lights off/active period). At the end of the feeding study, mice were euthanized by decapitation every 4 h at ZT 3, 7, 11, 15, 19, and 23. During the dark period (ZT 12–24), an infrared viewer was used for tissue collection at ZT 15, 19, and 23 with photic input to the suprachiasmatic nucleus (SCN) prevented by removing the eyes prior to light exposure (Filiano et al., 2013). Blood was collected in EDTA-treated tubes and placed on ice before centrifugation to obtain plasma. Livers were quickly divided into portions and placed into liquid nitrogen, RNA*later*™ stabilization solution (Invitrogen, Carlsbad, CA), or 10% buffered formalin. All procedures were approved by the UAB IACUC and compliant with the NIH Guide for the Care and Use of Laboratory Animals (8th ed., National Academy of Sciences, 2011).

### RNA Isolation and Real Time-PCR

Total RNA was isolated from liver using TRI-Reagent® (Sigma-Aldrich, St. Louis, MO). Isolated RNA was DNase treated with DNA-*free*™ DNase Treatment and Removal Reagents (Thermo Fisher Scientific, Waltham, MA) and the DNase-treated RNA (260/280 ratio > 1.8) was converted to cDNA using the High-Capacity cDNA Reverse Transcription Kit (Thermo Fisher Scientific, Waltham, MA). Measurements of mRNA levels were done by Real Time PCR (RT-PCR) using commercially available gene-specific TaqMan™ primers from Thermo Fisher Scientific (Waltham, MA) with either an Applied Biosystems 7900HT Real-time PCR or QuantStudio6 Flex Real-time PCR system (Thermo Fisher Scientific, Waltham, MA). Relative levels of mRNA for each gene were determined using the ΔΔCt method (Livak and Schmittgen, 2001) with values normalized to either glyceraldehyde-3-phosphate dehydrogenase (*Gapdh*) or peptidyl-prolyl isomerase A (*Ppia*). Data are presented as fold-change set to the control diet-Fl/Fl mice group trough (Udoh et al., 2017). A list of gene primers is provided in the supplemental data section (Supplementary Table 13).

### Plasma and Liver TG Measurements

Plasma TG was determined using reagents from Pointe Scientific (Canton, MI) and reported as mg TG/dL. For hepatic TG content, lipids were extracted by methods described previously (Filiano et al., 2013), TG was quantified using L-Type Triglyceride M colorimetric assay reagents (FUJIFILM Wako Diagnostics, Mountain View, CA) and reported as μg TG/mg liver.

### Liver Histopathology and Scoring

Liver was collected every 4 h at ZT 3, 7, 11, 15, 19, and 23 and a small piece of liver was dissected from the large outer right lobe of all mice, placed into 10% buffered formalin, and embedded in paraffin before being sectioned at a 5 μm thickness. Liver was mounted onto slides and stained with hematoxylin and eosin (H and E) according to accepted guidelines (Kleiner et al., 2005). Slides from each experimental group and for all time-points were coded with a unique number designation for unbiased examination and scoring by pathologists. Steatosis (percentage of hepatocytes containing lipid droplets), lobular inflammation (number of inflammatory foci), ballooning, and fibrosis were scored by pathologists blinded to the experimental design using the NAFLD activity scoring (NAS) protocol (Brunt et al., 1999). While the NAS protocol is not intended for ALD, we applied this system to assign a histopathology score to cases in this experimental animal study. Steatosis was scored as 0, <5%; 1, 5–33%; 2, >34–65%; and 3, >66% of hepatocytes containing lipid droplets. Lobular inflammation was scored as: 0, no foci; 1, <2 foci; 2, 2–4 foci, and 3, >4 foci. Ballooned hepatocytes were rare and fibrosis was undetectable; thus, histopathology scores reported in Table 1 largely reflect steatosis and inflammation. Pathologists also recorded the percentage of hepatocytes containing large and small droplet macrosteatosis.

**Table 1:** Statistical analyses of liver histopathology scores.

Histology variable	Control Fl/Fl mice (*N* = 35)	Ethanol Fl/Fl mice (*N* = 33)	Control LKO mice (*N* = 30)	Ethanol LKO mice (*N* = 31)	*p*
Histopathology score					
0	23 (65.7)^a^	22 (66.7)	20 (66.7)	12 (38.7)	0.020^b^
1	7 (20.0)	6 (18.2)	5 (16.7)	11 (35.5)	
2	2 (5.7)	4 (12.1)	3 (10.0)	3 (9.7)	
3	2 (5.7)	1 (3.0)	1 (3.3)	3 (9.7)	
4	1 (2.9)	0 (0)	0 (0)	1 (3.2)	
5	0 (0)	0 (0)	1 (3.3)	1 (3.2)	
Steatosis grade					
0 – <5%	33 (94.3)	27 (81.8)	27 (90.0)	16 (51.6)	0.660^c^
1 – 5–33%	1 (2.85)	6 (18.2)	3 (10.0)	12 (38.7)	0.016^d^
2 – 34–66%	1 (2.85)	0 (0)	0 (0)	3 (9.7)	
3 – >66%	0 (0)	0 (0)	0 (0)	0 (0)	
Lobular inflammation					
0 – none	20 (57.1)	24 (72.7)	21 (70.0)	19 (61.3)	0.314^e^
1 – <2	9 (25.7)	7 (21.2)	7 (23.3)	10 (32.3)	0.427^f^
2 – 2–4	5 (14.3)	1 (3.0)	0 (0)	1 (3.2)	
3 – >4	1 (2.9)	1 (3.0)	2 (6.7)	1 (3.2)	
Ballooning					
0 – none	35 (100)	33 (100)	27 (90)	29 (93.5)	0.093^g^
1 – few	0 (0)	0 (0)	3 (10)	3 (9.7)	0.108^h^
2 – many	0 (0)	0 (0)	0 (0)	0	

a Values are N (%).

b Values of *p* are from an ordinal contingency analysis for each parameter; significant Genotype X Diet Interaction.

c Values of *p* for steatosis are from a Fisher’s Exact Test comparing absence (steatosis grade 0) and presence (grade 1 + 2) in control-fed Fl/Fl vs. control-fed LKO mice.

d Values of *p* for steatosis are from a Fisher’s Exact Test comparing absence (steatosis grade 0) and presence (1 + 2) in alcohol-fed Fl/Fl vs. alcohol-fed LKO mice.

e Values of *p* for lobular inflammation are from a Fisher’s Exact Test comparing presence (1–3) or absence (0) of lobular inflammation in control-fed Fl/Fl vs. control-fed LKO mice.

f Values of *p* for lobular inflammation are from a Fisher’s Exact Test comparing presence (1–3) or absence (0) of lobular inflammation in alcohol-fed Fl/Fl vs. alcohol-fed LKO mice.

g Values of *p* for hepatocyte ballooning are from a Fisher’s Exact Test comparing presence or absence of ballooned hepatocytes in control-fed Fl/Fl vs. control-fed LKO mice.

h Values of *p* for hepatocyte ballooning are from a Fisher’s Exact Test comparing presence or absence of ballooned hepatocytes in alcohol-fed Fl/Fl vs. alcohol-fed LKO mice.

### Western Blotting

Livers were homogenized in 0.25 M sucrose buffer, pH 7.4, supplemented with protease and phosphatase inhibitor cocktails (Sigma-Aldrich, St. Louis, MO) and equal amounts of protein were separated on 4–20% Criterion™ TGX™ Precast Gels (Bio-Rad Laboratories, Hercules, CA) from all time-points of tissue collection (Udoh et al., 2015, 2017). Proteins were transferred from gels to nitrocellulose membranes and membranes were incubated at room temperature for 1 h in LI-COR® Blocking Buffer (LI-COR® Biosciences, Lincoln, NE). Membranes were incubated with primary antibodies overnight at 4°C: total ACC, 1:1,000 (cat. no. 3676, Cell Signaling Technologies, Danvers, MA); phospho-ACC-Ser79, 1:1,000 (cat. no. 11818, Cell Signaling Technology, Danvers, MA); FASN, 1:1,000 (cat no. 3180S, Cell Signaling Technology, Danvers, MA); and MCD, 1:1,000 (cat. no. PA5-22081, Thermo Fisher Scientific, Waltham, MA). An anti-β-actin antibody (cat. no. A5441, Sigma-Aldrich, St. Louis, MO) was used as the loading control (1:5,000). Membranes were washed and incubated in secondary antibodies from LI-COR® Biosciences (Lincoln, NE) for 1 h at room temperature. The secondary antibody used for total and phospho-ACC was IRDye 680RD (rabbit, cat. no. 926-68071), for MCD and FASN was 800CW (rabbit, cat. no. 926-32213), and for β-actin was 800CW (mouse, cat. no. 926-32210) or IRDye 680 RD (mouse, cat. no. 926-68070). Fluorescence was detected and densitometry of protein bands was quantified using the LI-COR Odyssey® CLx imaging system (Udoh et al., 2015, 2017). Data are presented as the average of protein densitometry across all time-points normalized to β-Actin for each treatment group as done in (Hatori et al., 2012). The antibodies used for total and phospho-ACC detect both ACC1 (265 kDa) and ACC2 (280 kDa) isoforms. Due to the close proximity of isoform bands on images, values represent the combined densitometry for total ACC1 and 2 and phospho-ACC1 and 2, respectively.

### Lipidomics

Lipidomics analysis was performed on liver lipid extracts collected at ZT 3 and ZT 15 from control and alcohol-fed Fl/Fl and LKO mice. Lipids were extracted from tissue using a modified Bligh and Dyer method (Bligh and Dyer, 1959). Briefly, 50–100 mg of frozen liver tissue was homogenized in 0.9 ml of dichloromethane and 2.0 ml of methanol (Honeywell Burdick and Jackson, Muskegon, MI). After homogenization, 10 μl of EquiSPLASH LIPIDOMIX Quantitative Mass Spec internal standard (cat. no. 330731, Avanti Polar Lipids, Alabaster, AL) was added and samples were incubated at room temperature for 30 min. One milliliter of liquid chromatography-mass spectrometry grade water (Honeywell Burdick and Jackson, Muskegon, MI) and 0.9 ml of dichloromethane were added, samples were centrifuged at 1200 rpm for 10 min at 4°C to separate phases, and the bottom hydrophobic organic phase containing lipids was collected and placed into a fresh glass test tube. The remaining upper hydrophilic phase was re-extracted with dichloromethane and the two organic phases were combined and dried under a stream of nitrogen. Lipids were re-solubilized in 300 μl injection solvent (dichloromethane:methanol, 50:50, with 5 mM ammonium acetate) and MS/MS^ALL^ mass spectrometric analysis and lipid species identification was performed as previously described in (Gajenthra Kumar et al., 2018) using a SCIEX Triple-TOF 6600+ (SCIEX, Framingham, MA) in the positive ion mode. Lipid species based on precursor fragment ion pairs were determined using the LipidView comprehensive target list (SCIEX, Framingham, MA) and the detected lipid species present in the TG pool (Supplementary Table 8) were compiled into a spreadsheet for statistical analyses. From these results, the percent TG FA saturation and percent TG FA composition were determined for the hepatic TG pool. The percent TG FA saturation was determined by dividing the sum of saturated fatty acids (SFA), monounsaturated fatty acids (MUFA), diunsaturated fatty acids (DUFA), or polyunsaturated fatty acids (PUFA) by the sum of all TG FA in the samples and multiplying by 100. Similarly, the percent TG FA composition was determined by dividing the sum of the most abundant individual TG FA species (12:0, 12:1, 14:0, 14:1, 16:0, 16:1, 18:1, 18:2, 20:0, 20:1, 20:2, 20:4, 22:1, and 22:6) by the sum of all TG FA in the samples and multiplying by 100.

### Statistical Analyses

Two or three-factor ANOVA, where indicated, were used to determine statistical significance of main effects of genotype, diet, time, and/or interactions with Tukey’s HSD test for *post hoc* analyses. To measure variation over time of day, cosinor analysis was performed to determine whether experimental measures fit a 24 h rhythm. For this, data were fitted to a cosine wave equation, f(*t*) = mesor +amplitude * cos[(2π*t*/*T*) + acrophase], in SPSS (IBM) using a non-linear regression module (Filiano et al., 2013; Udoh et al., 2015, 2017). The mesor (midline estimating statistic of rhythm) = mean of the rhythm; amplitude = 1/2 the distance between the peak and trough; *t* = time-point (ZT 3, 7, 11, 15, 19, or 23); *T* = the period (fixed to 24 h); and acrophase = ZT time of the peak of the rhythm, i.e., cosine maximum. Rhythmicity was determined using a linear regression model f(*t*) = M + cos (2π*t*/*T*) + sin (2π*t*/*T*), and data were considered rhythmic if the *p* of the *R*^2^ was ≤0.05. Student’s *t*-test was used to compare parameter estimates among the experimental treatment groups when the overall *R*^2^ was significant. Data that significantly fit a cosine function (rhythmic) are represented in graphs by solid lines, whereas non-significant data (arrhythmic) are represented by dashed lines. Sample sizes are included in figure legends with statistical significance set at *p* ≤ 0.05. Liver and plasma TG measurements were analyzed using non-parametric algorithm JTK_CYCLE in R (Hughes et al., 2010), with the period set to 24 h to test for rhythmicity in which statistical significance was set at *p* ≤ 0.05. Significant interactions of diet and genotype for the histopathology score was determined by ordinal contingency analysis comparing each treatment group, collapsed across ZTs. Steatosis grade, inflammation, and hepatocyte ballooning were assessed for significant differences in Fl/Fl vs. LKO control and alcohol-fed mice using Fisher’s Exact Test. For macrosteatosis, Fl/Fl control and LKO mice were compared across circadian time within diet groups. Hepatocytes containing small and large lipid droplets (macrosteatosis) were counted and quantified as a percentage of total hepatocytes in each image. Large droplet macrosteatosis percentages in both control diet groups were too low to warrant analysis. Distributions for large and small droplet macrosteatosis were positively skewed, which is often the case for count and percentage data. Positive skew for the small droplet macrosteatosis percentages within the alcohol group was corrected by a square root transformation. As a result, small droplet macrosteatosis data for alcohol-fed Fl/Fl and LKO mice were analyzed *via* cosinor analysis after square root transformation. For genotype comparisons of small droplet macrosteatosis percentages within the control diet group, transformation did not correct the positive skew. Thus, data were stratified by genotype and a negative binomial regression (Dupont, 2002) with linearized cosine functions as fixed factors [(Cos(2π*t*/24)) and Sin(cos(2π*t*/24))] was performed in SPSS (IBM) followed by planned *post hoc* comparisons between groups at each ZT. Likewise, large droplet macrosteatosis data were positively skewed and were analyzed similarly *via* negative binomial regression. For lipidomics analysis, we performed three-factor ANOVA to determine main effects of genotype, diet, time, and/or interactions with Tukey’s HSD for *post hoc* pairwise comparisons between each group. Two-factor ANOVA was performed for TG FA, where indicated, in cases that showed significant two-way interaction by three-factor ANOVA, but did not significantly show three-way interactions (Supplementary Tables 9–12).

## Results

This study is a follow-up from our previous work showing that alcohol and liver clock disruption differentially alter hepatic glycogen content and diurnal mRNA rhythms in glycogen and glucose metabolism genes (Udoh et al., 2017). In our previous paper, we validated the *Bmal1* LKO mouse model showing liver-specific absence of BMAL1 protein, and significant attenuation in transcript rhythms of *Bmal1* and the clock-controlled output gene D site of albumin promoter binding protein (*Dbp*) in the liver, whereas high amplitude mRNA rhythms in these genes and other clock genes were unaffected in extra-hepatic tissues (Udoh et al., 2017). Furthermore, we found no difference in alcohol intake, blood alcohol, and body weight gain between LKO and littermate control Fl/Fl mice (Udoh et al., 2017). Here, we extend our studies by investigating the combined effect of alcohol and liver clock disruption on temporal variations in diurnal mRNA rhythms in clock and lipid metabolism genes, hepatic and plasma TG levels, liver histopathology, and the FA composition of the hepatic TG pool.

### Circadian Clock Genes

**Figure 1:**
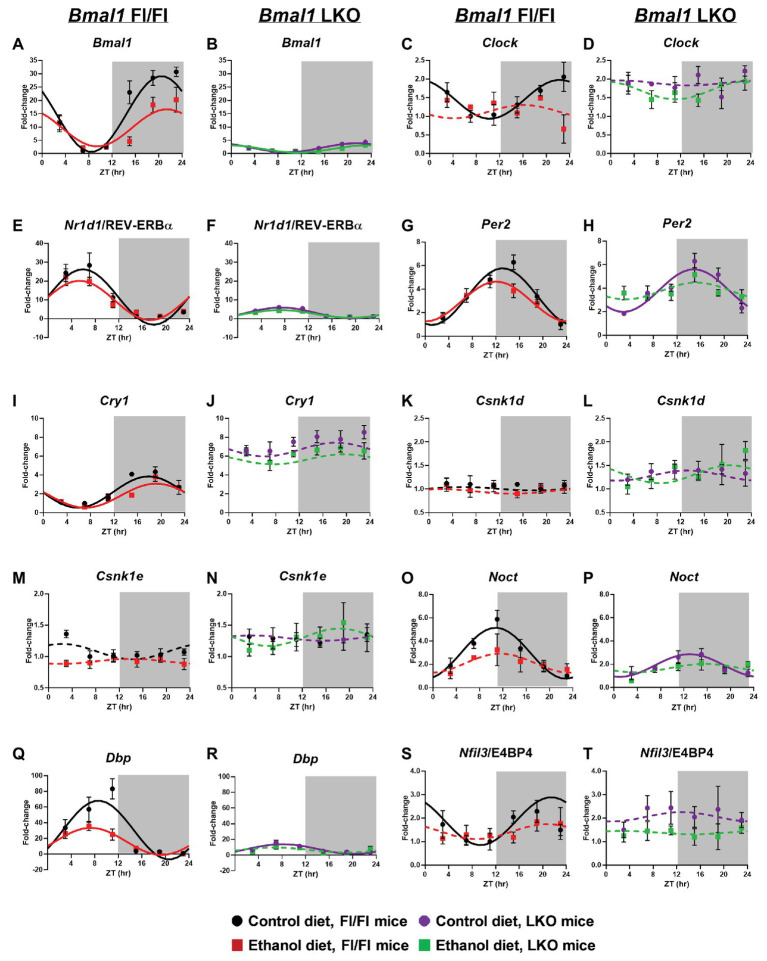
Chronic alcohol and liver clock disruption alter 24 h mRNA rhythms in molecular clock genes in the liver. Diurnal mRNA profiles of Brain and Muscle ARNT-like 1 (*Bmal1*; **A,B**), Circadian Locomotor Output Cycles Kaput (*Clock*; **C,D**), Nuclear Receptor Subfamily 1 Group D Member 1 (*Nr1d1*/Rev-Erbα; **E,F**), Period 2 (*Per2*; **G,H**), Cryptochrome 1 (*Cry1*; **I,J**), Casein Kinase 1 Delta (*Csnk1d*; **K,L**), Casein Kinase 1 Epsilon (*Csnk1e*; **M,N**), Nocturnin (*Noct*; **O,P**), D site of Albumin Promoter Binding Protein (*Dbp*; **Q,R**) and Nuclear Factor, Interleukin 3 Regulated (*Nfil3*/E4BP4; **S,T**) were measured in livers of control-fed (black) and alcohol-fed (red) *Bmal1* Flox/Flox (Fl/Fl) and control-fed (purple) and alcohol-fed (green) *Bmal1* liver-specific knockout (LKO) mice at ZT 3, 7, 11, 15, 19, and 23 (ZT 0: lights on/inactive period, ZT 12: lights off/active period, gray area) by real time (RT)-PCR. Data are presented as a fold-change to the *Bmal1* Fl/Fl control diet trough time-point and normalized to *Gapdh*. Cosinor analysis was performed using a nonlinear regression module in SPSS. Data are expressed as mean ± SEM for *n* = 4–8 mice/genotype/diet/time-point. Solid lines indicate rhythmic mRNA levels and a significant cosine fit, whereas dashed lines indicate arrhythmicity and a non-significant cosine fit. Results for Cosinor and ANOVA analyses are provided in Supplementary Tables 1 and 2, respectively.

Consistent with our previous findings (Filiano et al., 2013), chronic alcohol consumption significantly altered rhythmicity of clock gene mRNA levels in the liver (Cosinor analysis; Figure 1 and Supplementary Table 1). Specifically, alcohol decreased the midline estimating statistic of rhythm (mesor) and amplitude of *Bmal1* in liver of Fl/Fl mice (Figure 1A). A low amplitude *Bmal1* rhythm persists in livers of LKO mice that is most likely due to non-hepatocyte cell types in liver that do not express Cre recombinase (Figure 1B). Compared to control-fed mice, alcohol abolished rhythmic mRNA levels of the other positive regulator, *Clock*, in livers of Fl/Fl mice (Figure 1C). Diet had no effect on arrhythmic mRNA levels of *Clock* in livers from LKO mice (Figure 1D).

*Nr1d1*/REV-ERBα forms an important secondary feedback loop that regulates the clock *via Bmal1* transcriptional repression (Preitner et al., 2002). Rhythmic mRNA levels of *Nr1d1*/REV-ERBα were evident in livers from all four groups (Supplementary Table 1). In Fl/Fl mice, alcohol induced a significant ~3 h phase advance compared to control-fed mice (Figure 1E). Liver clock disruption significantly phase delayed and reduced the amplitude of *Nr1d1*/REV-ERBα rhythms in livers from LKO mice fed either diet compared to Fl/Fl mice (Figures 1E,F). LKO livers showed a ~5-fold reduction in *Nr1d1* amplitude compared to Fl/Fl mice (Figure 1F; Supplementary Table 1).

We next examined rhythmic transcription of *Per2* and *Cry1*, negative regulators of the clock (Kume et al., 1999; Zheng et al., 1999). Although alcohol had a minimal effect on diurnal rhythms in mRNA levels of *Per2* and *Cry1* in livers of Fl/Fl mice (Figures 1G,I), the combination of liver clock disruption and alcohol resulted in arrhythmic *Per2* mRNA levels (Figure 1H). *Per2* mRNA levels remained rhythmic in livers of control-fed LKO mice, albeit with a significant phase delay in the peak of *Per2* compared to control-fed Fl/Fl mice (Figure 1H). *Cry1* mRNA levels were arrhythmic in livers of LKO mice (Figure 1J).

Other factors that regulate the clock, or are themselves regulated by the clock, may also be affected by alcohol and/or liver clock disruption. For example, casein kinase 1 delta and epsilon (*Csnk1d* and *Csnk1e*) are two kinases that regulate clock timing through phosphorylation of core clock proteins (Vielhaber et al., 2000; Etchegaray et al., 2009; Lee et al., 2009). Measurements of mRNA levels for these genes were arrhythmic and unaffected by alcohol (Figures 1K–N). However, several important clock-controlled genes showed sensitivity to alcohol or the combination of alcohol and liver clock disruption. Both nocturnin (*Noct*; an mRNA deadenylase; Kojima et al., 2012) and *Dbp* (a D-box binding b-ZIP transcription factor) showed rhythmic mRNA levels in livers of control-fed mice; however, amplitude was significantly reduced in LKO mice compared to Fl/Fl mice (Figures 1O–R). Importantly, alcohol altered the diurnal rhythms in mRNA levels of *Noct* and *Dbp* in livers from mice of either genotype (Figures 1O–R). Although *Dbp* in livers of alcohol-fed Fl/Fl mice was still rhythmic, the amplitude was decreased by more than half (Figure 1Q). While control-fed LKO mice exhibited low amplitude *Dbp* and *Noct* rhythms, these rhythms were lost when LKO mice were fed alcohol (Figures 1P,R). Another D-box binding b-ZIP transcription factor, *Nfil3*/E4BP4 (interleukin 3 regulated/E4 promoter-binding protein 4; Mitsui et al., 2001; Ripperger and Schibler, 2006) displayed a significant diurnal rhythm in livers of control-fed Fl/Fl mice that was anti-phase to *Nr1d1*/REV-ERBα and *Dbp* (Figure 1S). Alcohol, liver clock disruption, or both, abolished *Nfil3*/E4BP4 rhythmicity (Figures 1S,T). Together, these results extend our previous findings and show that genetic disruption of the liver clock exacerbates the circadian rhythm-impairing effects of alcohol.

In order to assess whether alcohol-induced effects on overall clock mRNA levels depend on genotype (independent of time), we performed two-factor ANOVA on clock and clock-controlled genes (Supplementary Table 2). Alcohol reduced overall levels of *Bmal1*, *Clock*, *Cry1*, *Nfil3*/E4BP4, and *Dbp*, regardless of genotype (main effect of diet). In addition, livers from LKO mice generally had higher mRNA levels of *Clock*, *Per2*, *Cry1*, *Csnk1d*, and *Csnk1e*, as well as lower expression of *Bmal1*, *Dbp*, and *Nr1d1*/REV-ERBα, compared to livers from Fl/Fl mice (main effect of genotype). Although no Genotype X Diet interactions reached significance, there was a trend for *Bmal1*, *Dbp*, and *Nfil3*/E4BP4. Specifically, alcohol-fed Fl/Fl mice had lower mRNA levels of *Bmal1* and *Dbp*, whereas LKO mice on either diet had minimal levels of mRNA for these genes (Figures 1B,R). Higher *Nfil3*/E4BP4 mRNA levels in livers of control-fed LKO mice tended to be reduced when mice were fed alcohol (Figures 1S,T).

### Liver and Plasma TG Levels

**Figure 2:**
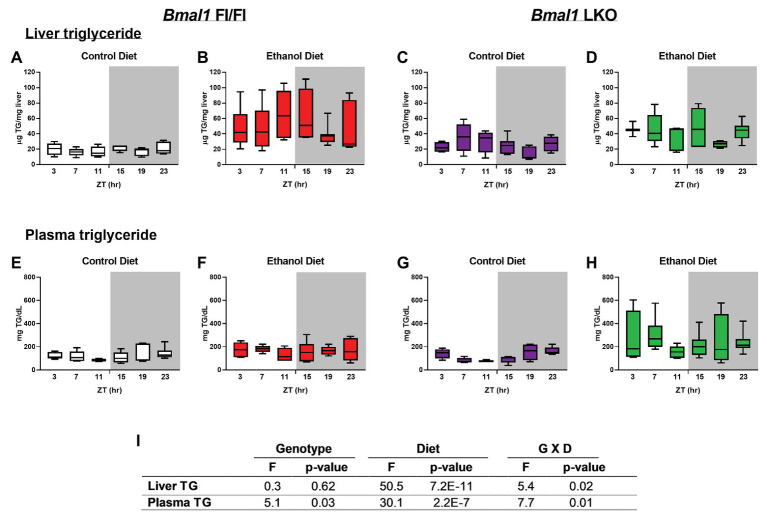
Chronic alcohol and liver clock disruption alter diurnal variations in liver and plasma triglyceride levels. Liver **(A–D)** and plasma **(E–F)** triglyceride (TG) were measured in samples collected from control-fed (**A,E** white) and alcohol-fed (**B,F** red) *Bmal1* Flox/Flox (Fl/Fl) and control-fed (**C,G** purple) and alcohol-fed (**D,H** green) *Bmal1* liver-specific knockout (LKO) mice at ZT 3, 7, 11, 15, 19, and 23 (ZT 0: lights on/inactive period, ZT 12: lights off/active period, gray area). Data are shown as min to max box and whisker plots for *n* = 3–8 mice/genotype/diet/time-point. Results from two-factor ANOVA are provided in **(I)**.

TG was significantly elevated in livers of alcohol-fed Fl/Fl and LKO mice (Figures 2B,D) compared to control diet counterparts (Figures 2A,C). Two-factor ANOVA showed significant interaction of Genotype X Diet for liver TG (Figure 2I). Plasma TG were also increased by alcohol in both genotypes (Figures 2F,H) compared to control diet mice (Figures 2E,G) with the highest overall levels of plasma TG observed in alcohol-fed LKO mice (Figure 2H). Plasma TG were rhythmic in control-fed Fl/Fl and LKO mice (Fl/Fl, *p* = 0.049; LKO, *p* = 0.0005; JTK cycle). Similar to liver TG, two-factor ANOVA for plasma TG shows a significant interaction of Genotype X Diet (Figure 2I). Taken together, these data illustrate differential impacts of liver clock disruption on TG metabolism in alcohol-fed mice.

### Liver Histopathology

A significantly greater percentage of livers from alcohol-fed LKO mice had higher overall histopathology scores (one or more) compared to the other three groups (Table 1). Steatosis was analyzed by comparing livers scored at grade 0 to those scored at grades 1 + 2 in control-fed and alcohol-fed mice. There was a statistically significant difference in steatosis grade in livers of alcohol-fed mice with increased numbers of livers exhibiting grade 1 + 2 compared to grade 0, and there was no effect in control-fed mice (Table 1). We observed no significant differences in lobular inflammation between groups. Ballooned hepatocytes were only seen in livers of LKO mice that were collected during the inactive period (ZT 0–12) of the day (Table 1). Mildly reactive Kupffer cells were also rare and only detected in livers of two alcohol-fed LKO mice.

**Figure 3:**
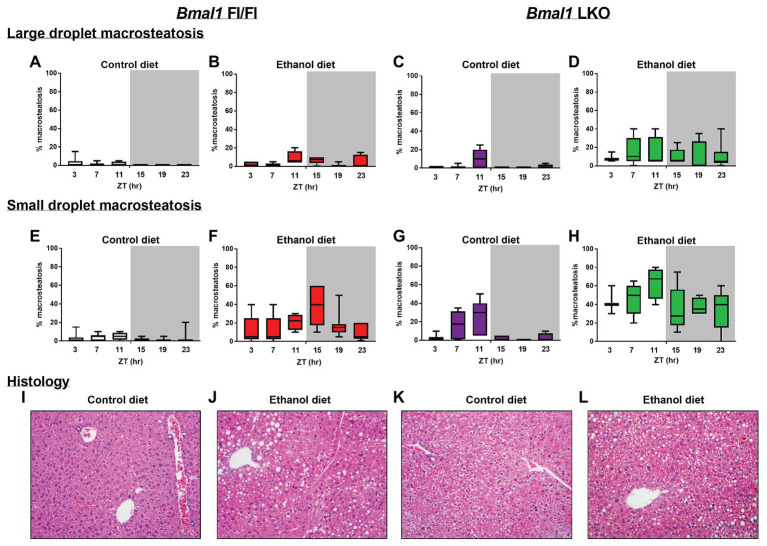
Chronic alcohol and liver clock disruption enhance liver histopathology. Large droplet **(A–D)** and small droplet **(E–H)** macrosteatosis were assessed in H and E-stained liver sections prepared from control-fed (**A,E** white) and alcohol-fed (**B,F** red) *Bmal1* Flox/Flox (Fl/Fl) and control-fed (**C,G** purple) and alcohol-fed (**D,H** green) *Bmal1* liver-specific knockout (LKO) mice at ZT 3, 7, 11, 15, 19, and 23 (ZT 0: lights on/inactive period, ZT 12: lights off/active period, gray area). Liver sections were scored at multiple magnifications. Data represent the percentage of hepatocytes containing large and small droplet macrosteatosis per section. Results shown as min to max box and whisker plots for *n* = 3–8 mice/genotype/diet/time-point. **(I–L)** Representative H and E stained histology images from each group at ZT 15 (20X mag). Results for statistical analyses are provided in Table 1.

We also examined livers for temporal differences in both large and small droplet macrosteatosis (Figure 3). Representative images are presented for livers collected at ZT 15 (Figures 3I–L). Both large (Figures 3A–D) and small droplet (Figures 3E–H) macrosteatosis were present; however, small droplet macrosteatosis was more prevalent with the highest levels seen in livers of alcohol-fed mice (Figures 3F,H). Low levels of large and small droplet macrosteatosis were observed in livers of some control-fed Fl/Fl mice (Figures 3A,E), with higher levels in livers of control-fed LKO mice (Figures 3C,G). In alcohol-fed mice, large droplet macrosteatosis displayed a significant 24 h rhythm (Figure 3B; *χ*^2^(5) = 12.8, *p* = 0.005) in livers of Fl/Fl mice but not in livers of LKO mice (Figure 3D; *χ*^2^(5) = 1.23, *p* = 0.805). Specifically, large droplet macrosteatosis in livers of alcohol-fed Fl/Fl mice were significantly less likely to be observed at ZT 3, 7, and 19 compared to ZT 11 (Figure 3B; *χ*^2^(5) = 12.7, *p* = 0.03). In LKO mice fed a control diet, livers had a decreased likelihood of large droplet macrosteatosis at ZT 3, 7, 15, 19, and 23 compared to ZT 11 (Figure 3C; *χ*^2^(5) = 53.6, *p* = 2.5E-10).

We also examined temporal dynamics of small droplet macrosteatosis. Cosinor analysis revealed that small droplet macrosteatosis exhibited a 24 h rhythm in livers of alcohol-fed Fl/Fl mice (Figure 3F; *R*^2^ = 0.29; *p* = 0.006; peak phase = ZT 14.44) but not in livers of alcohol-fed LKO mice (Figure 3H; *R*^2^ = 0.15; *p* = 0.11). In contrast, small droplet macrosteatosis was rhythmic in livers of control-fed LKO mice (Figure 3G; *χ*^2^(5) = 48.1, *p* = 3.3E-9), but not in livers of control-fed Fl/Fl mice (Figure 3E; *χ*^2^(5) = 8.0, *p* = 0.16). Livers of control-fed LKO mice had a decreased likelihood of small droplet macrosteatosis specifically at ZT 3, 15, 19, and 23 compared to ZT 7 and ZT 11 (Figure 3G; *χ*^2^(5) = 25.3, *p* < 0.05). Together, these results show that alcohol-induced macrosteatosis varies over the 24h day in a liver-clock dependent manner.

### Lipid Metabolism Transcription Factors

**Figure 4:**
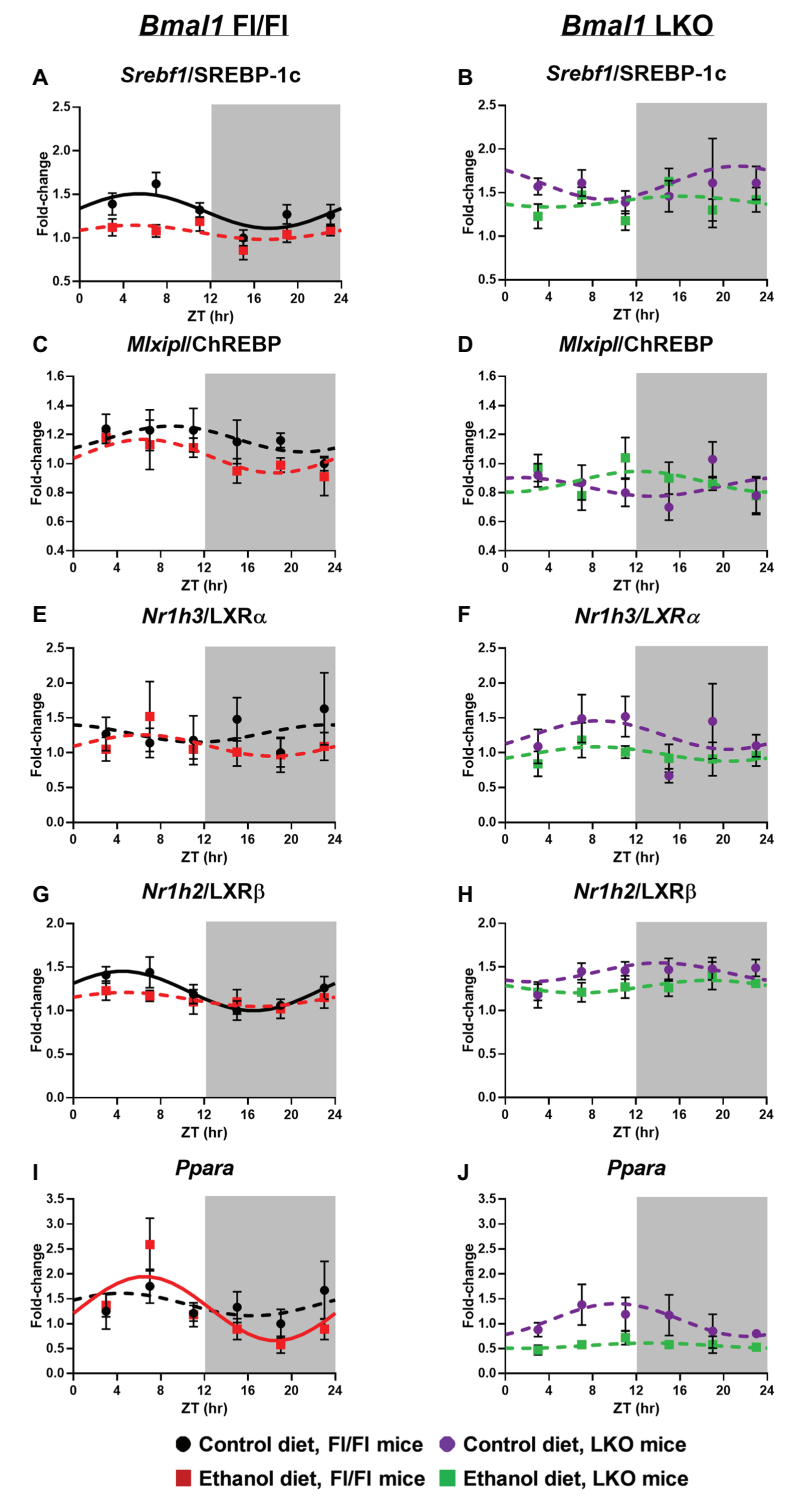
Chronic alcohol and liver clock disruption alter diurnal mRNA rhythms of lipid metabolism transcription factors in the liver. Diurnal mRNA profiles of Sterol regulatory element binding transcription factor 1 (*Srebf1*/SREBP-1C; **A,B**), MLX-interacting protein-like/Carbohydrate-responsive element-binding protein (*Mlxipl*/ChREBP; **C,D**), Nuclear receptor subfamily 1, group H, member 3/Liver X receptor alpha (*Nr1h3*/ LXRα; **E,F**), Nuclear receptor subfamily 1, group H, member 2/Liver X receptor beta (*Nr1h2*/LXRβ; **G,H**), Peroxisome proliferator-activated receptor alpha (*Pparα*; **I,J**) were measured in livers of control-fed (black) and alcohol-fed (red) *Bmal1* Flox/Flox (Fl/Fl) and control-fed (purple) and alcohol-fed (green) *Bmal1* liver-specific knockout (LKO) mice at ZT 3, 7, 11, 15, 19, and 23 (ZT 0: lights on/inactive period, ZT 12: lights off/active period, gray area) by RT-PCR. Data are presented as a fold-change to the *Bmal1* Fl/Fl control diet trough time-point and normalized to *Ppia*. Cosinor analysis was performed using a nonlinear regression module in SPSS. Data are expressed as mean ± SEM for *n* = 4–8 mice/genotype/diet/time point. Solid lines indicate rhythmic mRNA levels and a significant cosine fit, whereas dashed lines indicate arrhythmicity and a non-significant cosine fit. Results for Cosinor and ANOVA analyses are provided in Supplementary Tables 3 and 4, respectively.

Sterol regulatory element binding transcription factor 1/protein 1C (*Srebf1*/SREBP-1C) mRNA levels were rhythmic, peaking in the inactive period of the day in livers of control-fed Fl/Fl mice with alcohol inducing arrhythmicity (Figure 4A). *Srebf1*/SREBP-1C was arrhythmic in livers of LKO mice (Figure 4B). MLX interacting protein like/carbohydrate-responsive element binding protein (*Mlxipl*/ChREBP) was arrhythmic in all groups (Figures 4C,D). Nuclear receptor subfamily 1 group H member 3/Liver X receptor α (*Nr1h3*/LXRα) was also arrhythmic in all groups (Figures 4E,F), whereas *Nr1h2*/LXRβ was rhythmic in livers of control-fed Fl/Fl mice, but arrhythmic in livers of alcohol-fed Fl/Fl (Figure 4G) and LKO mice (Figure 4H). Peroxisome proliferator-activated receptor alpha (PPARα), a transcription factor that activates expression of many FAO and FA transport genes, was surprisingly arrhythmic in livers of control-fed Fl/Fl mice, but rhythmic in livers of alcohol-fed Fl/Fl mice (Figure 4I). *Ppara* was also arrhythmic in livers of LKO mice (Figure 4J). Results of cosinor analyses for lipid metabolism transcription factors are included in Supplementary Table 3.

Two-factor ANOVA was performed to determine significant main effects of alcohol and genotype on mRNA levels of lipid metabolism transcription factors, regardless of time (Supplementary Table 4). Independent of genotype, alcohol-fed mice generally displayed reduced mRNA levels of *Srebf1*/SREBP-1C, *Nr1h3*/LXRα, *Nr1h2*/LXRβ, and *Ppara*. Further, genetic disruption of the liver clock resulted in increased mRNA levels of *Srebf1*/SREBP-1C and *Nr1h2*/LXRβ, while decreasing mRNA levels of *Mlxipl*/ChREBP and *Ppara* (main effect of genotype). We also observed a trend toward a significant interaction of Genotype X Diet for *Mlxipl*/ChREBP.

### Fatty Acid Metabolism

**Figure 5:**
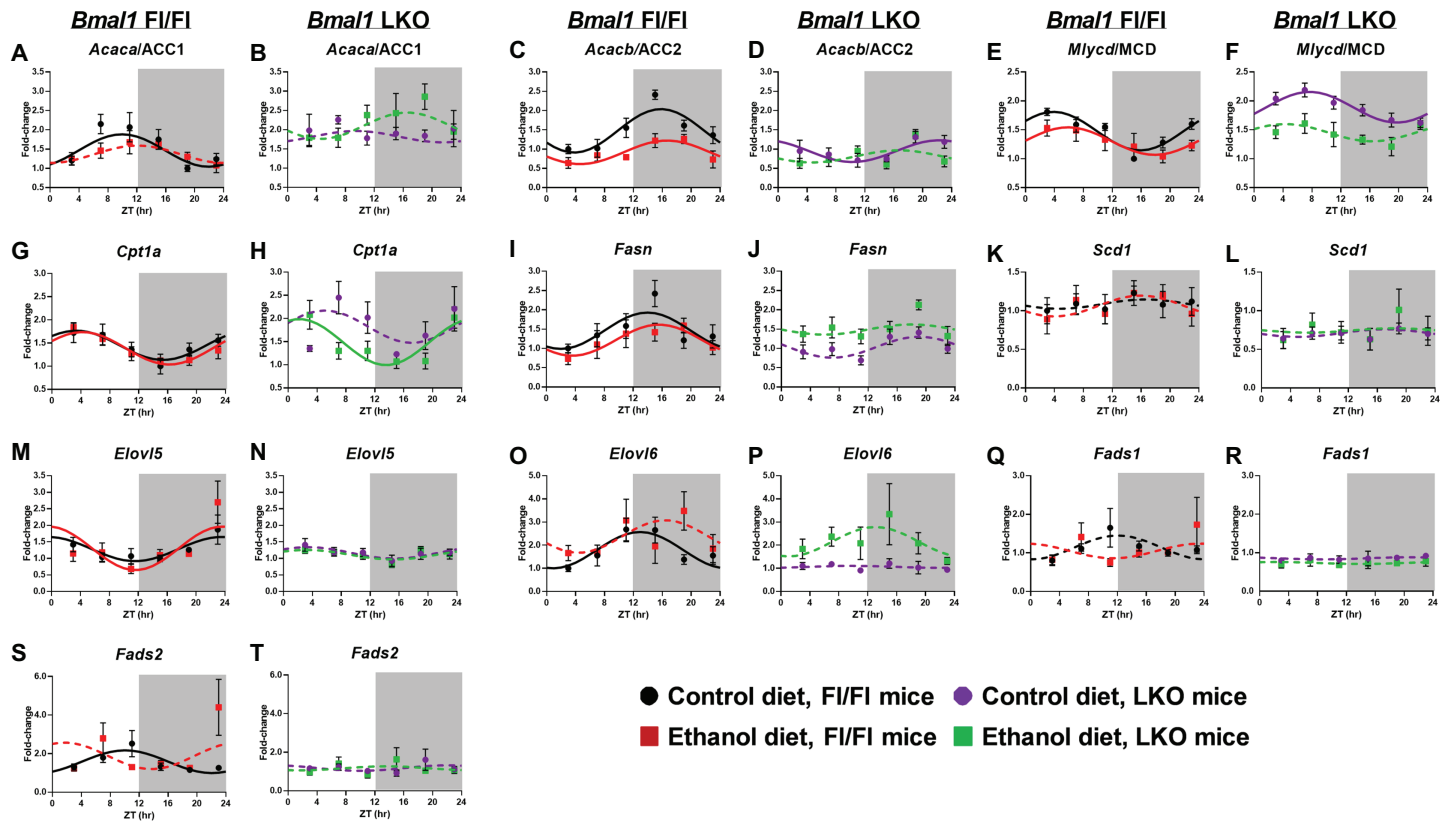
Chronic alcohol and liver clock disruption alter diurnal mRNA rhythms of fatty acid metabolism genes. Diurnal mRNA profiles of Acetyl-CoA carboxylase 1 (*Acaca*/ACC1; **A,B**), Acetyl-CoA carboxylase 2 (*Acacb*/ACC2; **C,D**), Malonyl-CoA decarboxylase (*Mlycd*/MCD; **E,F**), Carnitine-palmitoyl transferase 1A (*Cpt1a*; **G,H**), Fatty acid synthase (*Fasn*; **I,J**), Stearyl-CoA desaturase 1 (*Scd1*; **K,L**), Elongation of very long chain fatty acids protein 5 (*Elovl5*; **M,N**), Elongation of very long chain fatty acid protein 6 (*Elovl6*; **O,P**), Fatty acid desaturase 1 (*Fads1*; **Q,R**), and Fatty acid desaturase 2 (*Fads2*; **S,T**) were measured in livers of control-fed (black) and alcohol-fed (red) *Bmal1* Flox/Flox (Fl/Fl) and control-fed (purple) and alcohol-fed (green) *Bmal1* liver-specific knockout (LKO) mice at ZT 3, 7, 11, 15, 19, and 23 (ZT 0: lights on/inactive period, ZT 12: lights off/active period, gray area) by RT-PCR. Data are presented as a fold-change to the *Bmal1* Fl/Fl control diet trough time-point and normalized to *Ppia*. Data are expressed as mean ± SEM for *n* = 4–8 mice/genotype/diet/time point. Solid lines indicate rhythmic mRNA levels and a significant cosine fit, whereas dashed lines indicate arrhythmicity and a non-significant cosine fit. Results for Cosinor and ANOVA analyses are provided in Supplementary Tables 4 and 5, respectively.

Acetyl CoA carboxylase alpha/1 (*Acaca*/ACC1), an enzyme that catalyzes the formation of malonyl-CoA (substrate for *de novo* FA synthesis) by the carboxylation of acetyl-CoA (Thampy and Wakil, 1988), was rhythmic in livers of control-fed Fl/Fl mice and arrhythmic in livers of alcohol-fed Fl/Fl mice and LKO mice (Figures 5A,B). *Acacb*/ACC2 also catalyzes the generation of malonyl-CoA, which acts as an allosteric inhibitor of FAO by decreasing FA uptake into mitochondria by carnitine palmitoyltransferse-1A (CPT1A) activity (Abu-Elheiga et al., 2000). *Acacb*/ACC2 mRNA levels were rhythmic in livers of control and alcohol-fed Fl/Fl mice (Figure 5C) with alcohol significantly decreasing the mesor. While *Acacb*/ACC2 was rhythmic in livers of control-fed LKO mice (Figure 5D), the mesor was decreased and the peak was phase delayed in livers of control-fed LKO mice compared to Fl/Fl mice. *Acacb*/ACC2 was arrhythmic in livers of alcohol-fed LKO mice (Figure 5D). Malonyl CoA decarboxylase (*Mlycd*/MCD), an enzyme that catalyzes malonyl-CoA degradation to acetyl-CoA, was rhythmic in livers of control and alcohol-fed Fl/Fl mice (Figure 5E); however, alcohol feeding significantly decreased the mesor and induced a phase delay. *Mlycd*/MCD was rhythmic in livers of control-fed LKO mice (Figure 5F) with increased mesor and delayed peak mRNA levels compared to livers of control-fed Fl/Fl mice. *Mlycd*/MCD was arrhythmic in livers of alcohol-fed LKO mice (Figure 5F). *Cpt1a* was rhythmic in livers of Fl/Fl mice (Figure 5G) and alcohol-fed LKO mice (Figure 5H) with peak mRNA levels phase advanced in livers of alcohol-fed LKO mice compared to alcohol-fed Fl/Fl mice.

Fatty acid synthase (*Fasn*), a multi-functional enzyme that uses acetyl-CoA and malonyl-CoA to eventually produce palmitic acid (16:0), was rhythmic in livers of control and alcohol-fed Fl/Fl mice with peak mRNA levels occurring in the early active period (Figure 5I). *Fasn* was arrhythmic in livers of LKO mice (Figure 5J). FA elongation and biosynthesis of MUFA and PUFA is catalyzed through actions of stearoyl CoA desaturase 1 (*Scd1*), fatty acid desaturase 1 (*Fads1*), *Fads2*, and fatty acid elongase 5 (*Elovl5*) and *Elovl6* (Zhang et al., 2016a). *Scd1*, the rate-limiting step in the synthesis of MUFA, was arrhythmic in all groups (Figures 5K,L). *Elovl5* was rhythmic in livers of Fl/Fl mice (Figure 5M), but arrhythmic in livers of LKO mice (Figure 5N). Interestingly, diurnal rhythms in *Elovl6* mRNA levels were anti-phase to *Elovl5* mRNA levels when comparing control-fed Fl/Fl mice (Figures 5M,O) and was arrhythmic in livers of alcohol-fed Fl/Fl mice (Figure 5O) and livers of LKO mice (Figure 5P). *Fads1* mRNA levels were arrhythmic in all groups (Figures 5Q,R), whereas *Fads2* mRNA levels were rhythmic only in livers of control-fed Fl/Fl mice (Figures 5S,T). Results of cosinor analyses for these FA metabolism genes are provided in Supplementary Table 5.

Two-factor ANOVA (Supplementary Table 4) revealed that the overall mRNA levels of *Acaca*, *Acacb*, *Mlycd*, *Elovl6*, *Fads1*, and *Fads2* had significant main effects of genotype, independent of diet. In LKO mice, we observed overall increased mRNA levels of *Acaca*, *Mlycd*, and decreased *Acacb*, *Elovl6*, *Fads1*, *Fads2*, and *Scd1*. Independent of genotype, alcohol decreased overall mRNA levels of *Acacb* and *Mlycd* and increased mRNA levels of *Elovl6*. A significant interaction of Genotype X Diet was detected for *Acacb* where alcohol decreased *Acacb* in Fl/Fl, but not LKO mice. In contrast, alcohol increased *Fasn* mRNA levels in livers of LKO, but not Fl/Fl mice. In addition, a trend toward a significant interaction was observed for *Acaca* and *Mlycd*. Specifically, in LKO mice, mRNA levels of *Mlycd* tended to be decreased and *Acaca* mRNA levels increased by alcohol feeding.

**Figure 6:**
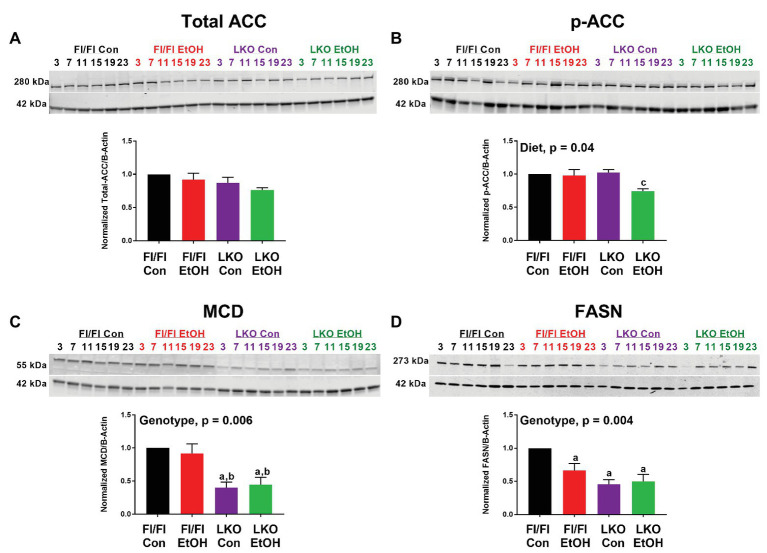
Chronic alcohol and liver clock disruption alter abundance and phosphorylation state of select fatty acid metabolism proteins. Diurnal protein abundances of total acetyl CoA carboxylase (Total ACC; **A**), phosphorylated acetyl CoA carboxylase (p-ACC; **B**), malonyl-CoA decarboxylase (MCD; **C**), and fatty acid synthase (FASN; **D**) were determined by western blotting using liver homogenates of control-fed (black) and alcohol-fed (red) *Bmal1* Flox/Flox (Fl/Fl) and control-fed (purple) and alcohol-fed (green) *Bmal1* liver-specific knockout (LKO) mice from ZT 3, 7, 11, 15, 19, and 23 (ZT 0: lights on/inactive period, ZT 12: lights off/active period). Protein abundances were detected using the LI-COR Odyssey^®^ digital imaging system. A representative image is presented for each protein of interest showing a full time course. The bar graphs indicate the average protein abundance calculated over the 24 h day normalized to β-actin, and levels are displayed as a fold-change from the control-fed Fl/Fl group. Data are shown as mean ± SEM *n* = 3–4 mice/genotype/diet. Significant two-factor ANOVA results are included in graphs. Letters indicate statistically significant differences (*p* < 0.05) for comparisons to control-fed Fl/Fl mice (a), alcohol-fed Fl/Fl mice (b), and control-fed LKO mice (c).

We also examined protein abundance and phosphorylation for a small set of FA metabolism enzymes, ACC, MCD, and FASN, to complement gene expression analyses. As no time-of-day differences were detected, results were analyzed by pooling all time points for each treatment group, as done in previous studies (Hatori et al., 2012). While the antibodies used for total and phospho-ACC detect both ACC1 (265 kDa) and ACC2 (280 kDa) isoforms, our data largely showed the presence of the lower molecular weight lipogenic ACC1 isoform in liver (Figure 6), which is in agreement with other studies in rodents (Bianchi et al., 1990). Total ACC was unchanged in all treatment groups (Figure 6A), whereas p-ACC levels were significantly lower in livers of alcohol-fed LKO mice compared to levels measured in control-fed LKO mice (Figure 6B). Two-factor ANOVA showed a significant main effect of diet for p-ACC and a trend toward a significant Genotype X Diet interaction (*p* = 0.07). MCD was significantly lower in livers of LKO mice compared to Fl/Fl mice (Figure 6C) with a significant effect of genotype. Similarly, FASN was lower in livers of alcohol-fed Fl/Fl mice and LKO mice compared to control-fed Fl/Fl mice (Figure 6D). Two-factor ANOVA showed a significant genotype effect for FASN.

### Triglyceride Metabolism

**Figure 7:**
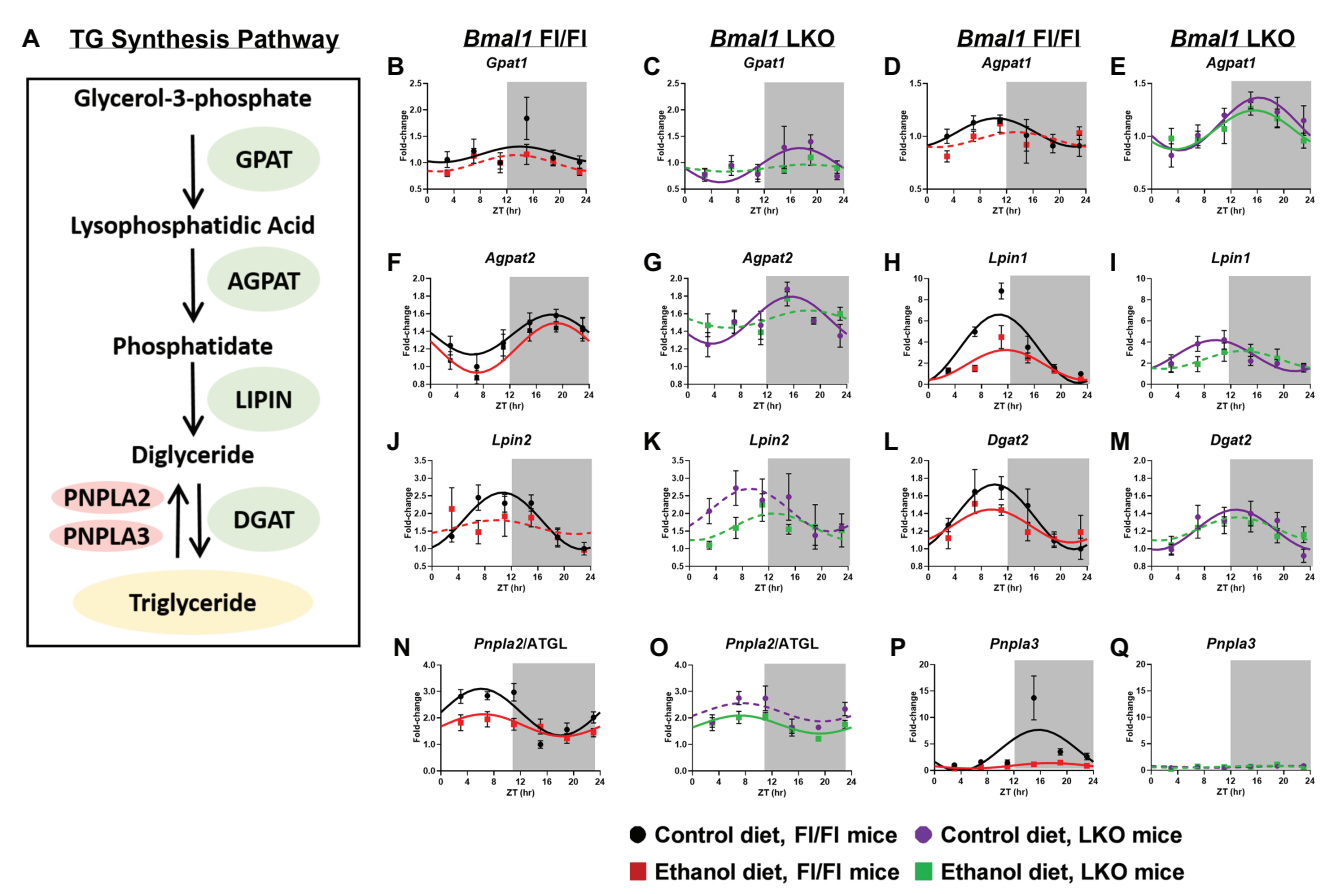
Chronic alcohol and liver clock disruption alter diurnal mRNA rhythms of triglyceride metabolism genes. Pathway for triglyceride synthesis/breakdown **(A)**. Diurnal mRNA profiles of Glycerol-3-phosphate acyltransferase (*Gpat1*; **B,C**), 1-acyl-sn-glycerol-3-phosphate acyltransferase alpha (*Agpat1*; **D,E**), 1-acyl-sn-glycerol-3-phosphate acyltransferase beta (*Agpat2*; **F,G**), Lipin-1 (*Lpin1*; **H,I**), Lipin-2 (*Lpin2*; **J,K**), Diacylglycerol o-acyltransferase 2 (*Dgat2*; **L,M**), Patatin-like phospholipase domain-containing protein 2/Adipose Triglyceride Lipase (*Pnpla2*/ATGL; **N,O**), Patatin-like phospholipase domain-containing protein 3 (*Pnpla3*; **P,Q**) were measured in livers of control-fed (black) and alcohol-fed (red) *Bmal1* Flox/Flox (Fl/Fl) and control-fed (purple) and alcohol-fed (green) *Bmal1* liver-specific knockout (LKO) mice at ZT 3, 7, 11, 15, 19, and 23 (ZT 0: lights on/inactive period, ZT 12: lights off/active period, gray area) by RT-PCR. Data are presented as a fold-change to the *Bmal1* Fl/Fl control diet trough time-point and normalized to *Ppia*. Data are expressed as mean ± SEM for *n* = 4–8 mice/genotype/diet/time point. Solid lines indicate rhythmic mRNA levels and a significant cosine fit, whereas dashed lines indicate arrhythmicity and a non-significant cosine fit. Results for Cosinor and ANOVA analyses are provided in Supplementary Tables 4 and 6, respectively.

Previous studies report diurnal rhythms in mRNA levels of TG metabolism genes (Figure 7A) in the liver (Adamovich et al., 2014); however, the impact alcohol and liver clock disruption have on these day/night differences is unknown. Glycerol-3-phosphate acyltransferase 1 (*Gpat1*), the first enzyme of TG synthesis that catalyzes acylation of glycerol-3-phosphate (G3P) to lysophosphatidic acid (LPA; Coleman and Mashek, 2011), was rhythmic in livers of control-fed Fl/Fl and LKO mice, whereas mRNA levels were arrhythmic in livers of alcohol-fed mice (Figures 7B,C). The next step in TG synthesis involves 1-acyl-sn-glycerol-3-phosphate acyltransferase 1 and 2 (AGPAT1, 2) that transfer an additional FA to LPA producing phosphatidate (PA). *Agpat1* was rhythmic in livers of both control-fed Fl/Fl and LKO mice (Figures 7D,E) with peak *Agpat1* mRNA levels phase delayed in livers of LKO mice. *Agpat1* was arrhythmic in livers of alcohol-fed Fl/Fl mice, but rhythmic in livers of alcohol-fed LKO mice (Figures 7D,E). *Agpat2* mRNA levels were rhythmic in livers of Fl/Fl mice (Figure 7F), as well as livers of control-fed LKO mice, but not livers of alcohol-fed LKO mice (Figure 7G). The peak of *Agpat2* was phase advanced ~3.5 h in livers of control-fed LKO compared to control-fed Fl/Fl mice. Lipins catalyze diglyceride (DG) formation through phosphatidate phosphatase-1 activity (Reue and Wang, 2019). *Lpin1* displayed a high amplitude rhythm in livers of control-fed Fl/Fl mice that was significantly lower in livers of alcohol-fed Fl/Fl mice (Figure 7H) and control-fed LKO mice (Figure 7I). *Lpin1* was arrhythmic in livers of alcohol-fed LKO mice (Figure 7I). *Lpin2* mRNA levels were also rhythmic in livers of control-fed Fl/Fl mice, but arrhythmic in livers of alcohol-fed Fl/Fl mice (Figure 7J) and LKO mice (Figure 7K). The last step of TG synthesis is the conversion of DG to TG by diacylglycerol O-acyltransferase (DGAT) enzymes. *Dgat2* mRNA levels were rhythmic in livers of control and alcohol-fed Fl/Fl mice (Figure 7L) and control-fed LKO mice (Figure 7M), but arrhythmic in livers of alcohol-fed LKO mice (Figure 7M).

We also measured 24 h rhythms in mRNA levels for patatin like phospholipase domain containing 2/adipose triglyceride lipase (*Pnpla2*/ATGL) and *Pnpla3*, which are lipases that mediate the first step in TG breakdown (Figure 7A; Trepo et al., 2016; Schreiber et al., 2019). *Pnpla2*/ATGL mRNA levels were rhythmic in livers of both control-fed and alcohol-fed Fl/Fl mice; however, alcohol feeding significantly decreased the mesor and amplitude of *Pnpla2*/ATGL (Figure 7N). Lower mRNA levels of *Pnpla2*/ATGL were also seen in livers of control-fed LKO mice with alcohol inducing arrhythmicity (Figure 7O). *Pnpla3* exhibited a high amplitude rhythm in livers of control-fed Fl/Fl mice that was significantly decreased by alcohol feeding (Figure 7P). *Pnpla3* was arrhythmic in livers of LKO mice (Figurer 7Q). Cosinor analyses results for TG metabolism genes are included in Supplementary Table 6.

Assessment of mRNA levels of TG metabolism genes was next performed by two-factor ANOVA to determine whether the observed effects of alcohol were dependent on genotype, regardless of time of day (Supplementary Table 4). Alcohol decreased mRNA levels of *Agpat1*, *Lpin1*, *Lpin2*, and *Pnpla2*/ATGL and *Pnpla3*. Significant main effects of genotype were found for *Agpat1* and *Agpat2*, which were generally increased in livers of LKO mice. In contrast, *Pnpla3* mRNA levels were significantly decreased in livers of LKO mice. We also observed a significant interaction of Genotype X Diet for *Pnpla3*, due to the dramatic alcohol-induced decrease in *Pnpla3* mRNA levels observed in Fl/Fl mice.

### Lipid Droplet-Associated Components

**Figure 8:**
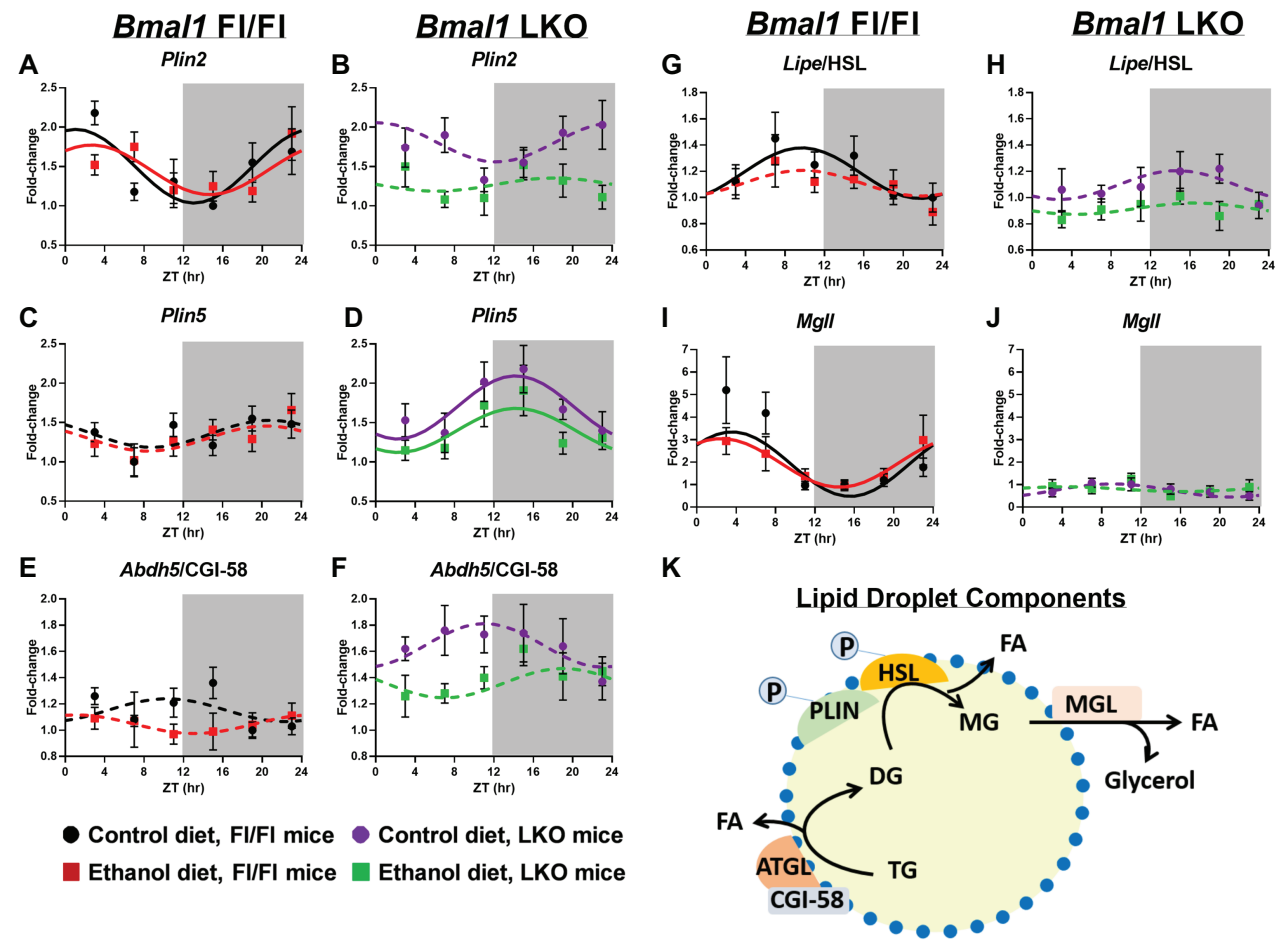
Chronic alcohol and liver clock disruption alter diurnal mRNA rhythms of lipid droplet-associated genes. Diurnal mRNA profiles of lipid droplet genes Perilipin 2 (*Plin2*; **A,B**), Perilipin 5 (*Plin5*; **C,D**), Abhydrolase domain containing 5/Comparative Gene Interaction-58 (*Abdh5*/CGI-58; **E,F**), Hormone sensitive lipase (*Lipe*/HSL; **G,H**), and Monoacylglycerol lipase (*Mgll*; **I,J**) were measured in livers of control-fed (black) and alcohol-fed (red) *Bmal1* Flox/Flox (Fl/Fl) and control-fed (purple) and alcohol-fed (green) *Bmal1* liver-specific knockout (LKO) mice at ZT 3, 7, 11, 15, 19, and 23 (ZT 0: lights on/inactive period, ZT12: lights off/active period, gray area) by RT-PCR. Data are presented as a fold-change to the *Bmal1* Fl/Fl control diet trough time-point and normalized to *Ppia*. Data are expressed as mean ± SEM for *n* = 4–8 mice/genotype/diet/time point. Solid lines indicate rhythmic mRNA levels and a significant cosine fit, whereas dashed lines indicate arrhythmicity and a non-significant cosine fit. Diagram of a lipid droplet and lipid droplet-associated turnover proteins and enzymes **(K)**. After phosphorylation, PLIN recruits lipases to the surface of the lipid droplet to initiate TG breakdown to glycerol and FA. Results for Cosinor and ANOVA analyses are provided in Supplementary Tables 4 and 7, respectively.

Lipid droplet turnover is regulated by the coordinated actions of multiple proteins located on the lipid droplet surface (Figure 8K). First, we measured 24 h rhythms in mRNA levels of two members of the perilipin (PLIN) family, *Plin2* and *Plin5*, which recruit lipases to the lipid droplet surface initiating lipolysis (Itabe et al., 2017). *Plin2* mRNA levels were rhythmic in livers of Fl/Fl mice (Figure 8A) and arrhythmic in LKO mice (Figure 8B). In contrast, *Plin5* was arrhythmic in livers of Fl/Fl mice (Figure 8C), but rhythmic in livers of LKO mice (Figure 8D). Abhydrolase domain containing 5/comparative gene identification-58 (*Abdh5*/CGI-58), postulated to activate *Pnpla2*/ATGL and start TG hydrolysis (Lass et al., 2006), was arrhythmic in all groups (Figures 8E,F). Lipase E/hormone sensitive type (*Lipe*/HSL), which catalyzes DG hydrolysis, was rhythmic in livers of control-fed Fl/Fl mice, but arrhythmic in livers of alcohol-fed Fl/Fl mice (Figure 8G) and livers of LKO mice (Figure 8H). The final step in lipolysis is catalyzed by monoacylglycerol lipase (*Mgll*), which works in concert with *Lipe*/HSL to hydrolyze DG to FA and glycerol (Fredrikson et al., 1986). *Mgll* was rhythmic in livers of control and alcohol-fed Fl/Fl mice (Figure 8I), but arrhythmic in livers of LKO mice (Figure 8J). Results of cosinor analyses for lipid droplet-associated genes are included in Supplementary Table 7.

We examined genotype and alcohol-induced effects on mRNA levels of lipid droplet components by two-factor ANOVA, independent of time (Supplementary Table 4). Livers of LKO mice generally exhibited increased mRNA levels of *Plin5* and *Abdh5*/CGI-58 and decreased *Lipe*/HSL and *Mgll* (main effect of genotype). Alcohol decreased mRNA levels of *Plin2*, *Plin5*, *Lipe/*HSL, and *Abdh5*/CGI-58, independent of genotype (main effect of diet). *Plin2* mRNA levels were lower in livers of alcohol-fed LKO mice, with a significant Genotype X Diet interaction. A trend toward a significant Genotype X Diet interaction was found for *Plin5* and *Abdh5*/CGI-58, where increased mRNA levels of these genes in livers of LKO mice tended to be decreased by alcohol.

### Lipidomics

**Figure 9:**
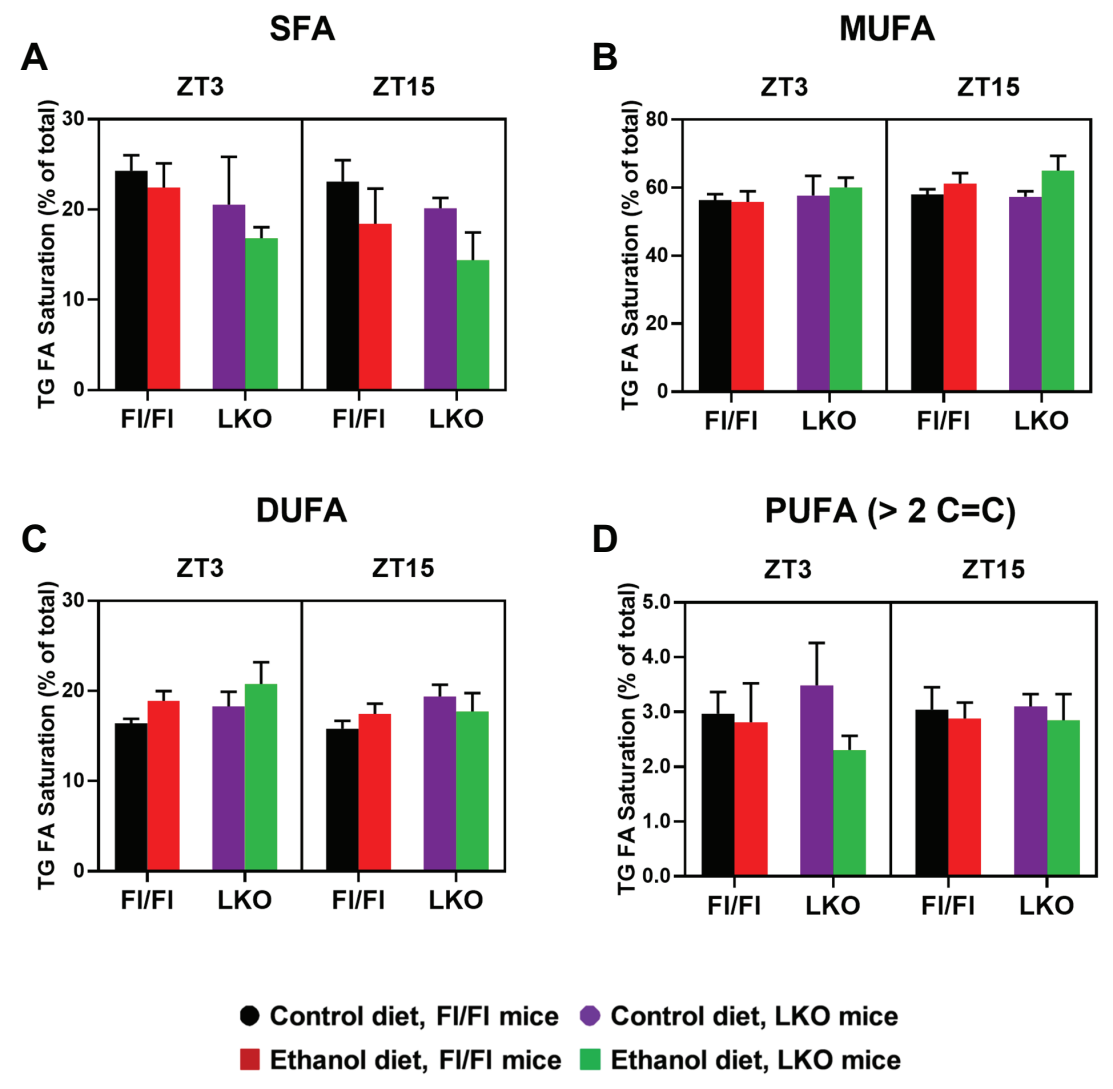
Chronic alcohol and liver clock disruption differentially alter triglyceride fatty acid saturation in the liver. Triglyceride (TG) fatty acid (FA) saturation as a percentage of total saturated fatty acids (SFA, **A**), monounsaturated fatty acids (MUFA, **B**), diunsaturated fatty acids (DUFA, **C**), and polyunsaturated fatty acids (PUFA, **D**) was determined in liver lipid extracts from control-fed (black) and alcohol-fed (red) *Bmal1* Flox/Flox (Fl/Fl) and control-fed (purple) and alcohol-fed (green) *Bmal1* liver-specific knockout (LKO) mice at ZT 3 and ZT 15 (ZT = 0: lights on/inactive active, ZT = 12: lights offs/active period) using MS/MS^ALL^. Data are mean ± SEM for *n* = 3–4 mice/genotype/diet/time-point. Results for ANOVA analyses are provided in Supplementary Table 9.

Finally, to gain greater insight into the effects alcohol and liver clock disruption have on lipid metabolism we conducted a lipidomics analysis on livers collected at ZT 3 and ZT 15. A complete list of the TG species detected in livers is provided in Supplementary Table 8 and these results were used to calculate the percent TG FA saturation and percent TG FA composition in the hepatic TG pool. First, we analyzed for effects on FA saturation. Hepatic TG FA were grouped accordingly to the saturation state (SFA, MUFA, DUFA, or PUFA) and data were analyzed by three-factor ANOVA (Supplementary Table 9). Alcohol significantly decreased the percentage of TG SFA in livers of Fl/Fl and LKO mice compared to control diet mice (Figure 9A) with significant main effects of genotype and diet. In contrast, TG MUFA are increased in livers of alcohol-fed mice (vs. control-fed mice) and at ZT 15 (vs. ZT 3; Figure 9B) with significant main effects of diet and time. Alcohol also increased TG DUFA levels (compared to control-fed mice) and livers from LKO mice had higher TG DUFA than livers from Fl/Fl mice (main effects of genotype and diet; Figure 9C). TG PUFA made up the smallest percentage of FA detected in the hepatic TG pool. Alcohol decreased the percentage of TG PUFA (main effect of diet; Figure 9D).

**Figure 10:**
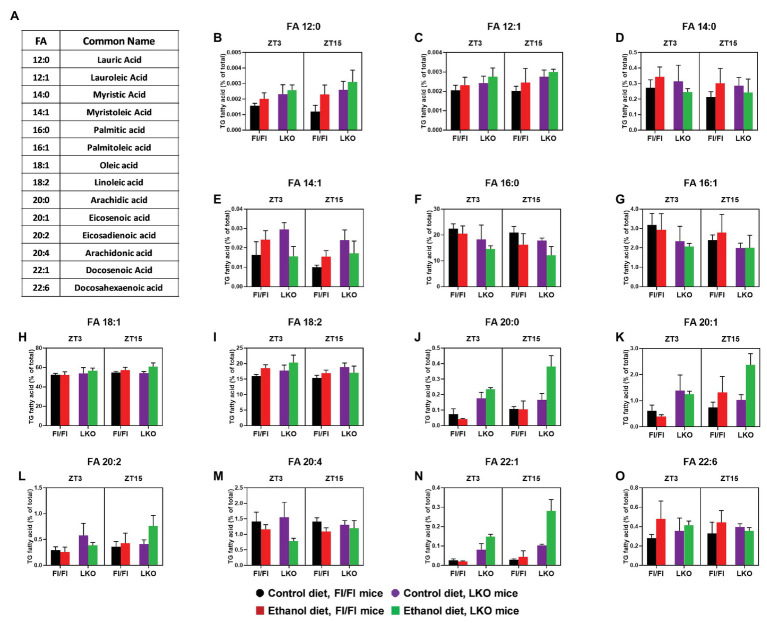
Chronic alcohol and liver clock disruption differentially alter triglyceride fatty acid composition in the liver. Fatty acids (FA, **B–O**) as a percentage of the total triglyceride (TG) FA pool was determined in liver lipid extracts from control-fed (black) and alcohol-fed (red) *Bmal1* Flox/Flox (Fl/Fl) and control-fed (purple) and alcohol-fed (green) *Bmal1* liver-specific knockout (LKO) mice at ZT 3 and ZT 15 (ZT 0: lights on/inactive period, ZT 12: lights offs/active period) using MS/MS^ALL^. FA common names and structure are shown in panel **(A)**. Data are mean ± SEM for *n* = 3–4 mice/genotype/diet/time-point. Results for ANOVA analyses and relevant pair-wise comparisons are provided in Supplementary Tables 9–12, respectively.

Next, we analyzed lipidomics results to look for effects on the individual FA species in the hepatic TG pool (Figure 10A). Results for three-factor ANOVA are provided in Supplementary Table 9. The significant interaction of Genotype X Diet X Time was observed for several long chain FA with 20 or more carbons. Specifically, pair-wise comparisons (Supplementary Table 10) show that the combination of alcohol and liver clock disruption significantly increased liver content of arachidic acid (20:0; Figure 10J), eicosadienoic acid (20:2; Figure 10L), and docosenoic acid (22:1; Figure 10N), especially at ZT 15. In contrast, alcohol and liver clock disruption significantly decreased arachidonic acid (20:4), specifically at ZT 3 (Figure 10M).

Significant main effects of genotype, diet, or time were also found for several FA species. Independent of genotype and time (main effect of diet; Supplementary Table 9), alcohol significantly increased lauric acid (12:0; Figure 10B), lauroleic acid (12:1; Figure 10C), and the most abundant FA, oleic acid (18:1; Figure 10H). The PUFA docosahexanoic acid (22:6) also tended to be increased in livers of alcohol-fed mice compared to control-fed mice (Figure 10O). Interestingly, alcohol induced an overall decrease in palmitic acid (16:0), the enzymatic product of FASN and a long chain FA precursor, compared to mice fed the control diet (Figure 10F). Genetic disruption of the liver clock alone (main effect of genotype; Supplementary Table 9) induced an overall increase in lauric acid (12:0; Figure 10B), lauroleic acid (12:1; Figure 10C) and linoleic acid (18:2; Figure 10I). We also observed a general decrease in the levels of palmitic acid (16:0; Figure 10F) in livers of LKO mice compared to Fl/Fl mice (main effect of genotype; Supplementary Table 9). A time-of-day difference was observed for the overall level of myristoleic acid (14:1; Figure 10E), which was higher at ZT 3 regardless of genotype or diet (main effect of time; Supplementary Table 9).

Where significant two-factor interactions (Genotype X Diet or Diet X Time) and no three-factor interaction was present, we conducted two-factor ANOVA with pairwise comparisons between groups. Specifically, corresponding data were collapsed together across time for a Genotype X Diet interaction or across genotype for a Diet X Time interaction. No Genotype X Time interactions were present. These additional analyses revealed that independent of time there was a significant interaction of Genotype X Diet (Supplementary Tables 9 and 11) for myristic acid (14:0; Supplementary Figure 1A) and myristoleic acid (14:1 Supplementary Figure 1B). Alcohol significantly increased overall levels of myristic acid (14:0) in livers of Fl/Fl mice (Supplementary Figure 1A); however, pair-wise comparisons did not reach significance (Supplementary Figure 1C). Pair-wise comparison revealed increased levels of myristoleic acid (14:1) in livers of control-fed LKO mice compared to control-fed Fl/Fl mice and alcohol-fed LKO mice (Supplementary Figures 1B,C). Time-of-day dependent effects of alcohol feeding (significant interaction of Diet X Time; Supplementary Table 8) were observed for linoleic acid (18:2) and eicosenoic acid (20:1), regardless of genotype. Specifically, at ZT 3 alcohol-fed mice have increased linoleic acid levels, whereas alcohol feeding has no significant effect at ZT 15 (Supplementary Figures 2A,C). Conversely, higher levels of eicosenoic acid are present in livers of alcohol-fed mice compared to control-fed mice at ZT 15, but not ZT 3 (Supplementary Figures 2B,C). Collectively, these exciting new lipidomics findings indicate that chronic alcohol consumption and liver clock disruption differentially affect FA saturation and composition of the hepatic TG pool.

## Discussion

In this study, we provide a temporal analysis of hepatic lipid metabolism at multiple levels (mRNA, protein abundance, metabolite, and histology) in livers of mice with an intact (Fl/Fl) or disrupted liver clock (LKO) fed either a control or alcohol-containing diet. Several novel findings regarding lipid metabolism were revealed in these studies. First, small droplet macrosteatosis and plasma TG are higher in alcohol-fed LKO mice compared to Fl/Fl mice. Second, LKO mice exhibit different time-of-day patterns of hepatic TG and steatosis type compared to Fl/Fl mice. Third, alcohol or clock disruption alone significantly alters 24 h rhythms in mRNA levels of clock and lipid metabolism genes, with some genes differentially impacted by alcohol in livers of LKO mice. Finally, lipidomics revealed novel changes in TG FA content in livers of alcohol-fed and clock-disrupted mice in a time-of-day dependent manner. In summary, these new findings show that the liver clock is important for maintaining diurnal control of lipid metabolism and that disrupting the liver clock exacerbates alcohol-related hepatic steatosis.

Previously, we found that alcohol consumption disrupts 24 h rhythms in mRNA levels of clock genes and induces circadian misalignment between the master clock in the SCN of the hypothalamus and the peripheral liver clock in wild-type mice (Filiano et al., 2013). These findings were replicated in subsequent alcohol studies from other laboratories (Zhou et al., 2014; Zhang et al., 2018). In this current paper, we observed similar changes in rhythmic mRNA levels of clock genes in livers of alcohol-fed Fl/Fl mice. Alcohol dampened diurnal rhythms in mRNA levels of *Bmal1*, *Per2*, and *Nr1d1*/REV-ERBα and the clock-controlled transcription factor *Dbp*. Interestingly, alcohol induced arrhythmic *Clock*, *Noct*, and *Nfil3*/E4BP4 mRNA levels. A recent RNA-seq analysis from global *Nfil3*/E4BP4 knockout mice shows that this b-ZIP transcription factor represses liver mRNA rhythms (Yoshitane et al., 2019), suggesting that *Nfil3*/E4BP4 (a repressor) might work in concert with BMAL1 (an activator) to temporally regulate liver metabolism. While *Nfil3*/E4BP4 has mostly been studied in immune cells (Male et al., 2012), emerging work points to a pivotal role of *Nfil3*/E4BP4 in regulating liver metabolism, including *de novo* lipogenesis (Tong et al., 2016; Zheng et al., 2016), gluconeogenesis (Kang et al., 2017), and FGF21 (Tong et al., 2010, 2013) – pathways implicated in ALD.

For the first time, the present study also assessed 24 h mRNA levels of clock genes in mice with a non-functional liver clock – LKO mice (Udoh et al., 2017). As expected, we observed arrhythmic or dampened mRNA levels for most of the clock and clock-controlled genes in livers of LKO mice compared to Fl/Fl mice, with alcohol further decreasing rhythmic amplitude. We also observed differential effects on mRNA levels of clock genes between genotypes. For example, *Bmal1*, *Nr1d1*/REV-ERBα, *Dbp*, and *Noct* have a lower mesor in livers of LKO mice compared to Fl/Fl mice. Conversely, *Clock*, *Cry1*, and *Csnk1d/e*, while arrhythmic in livers of LKO mice, have higher overall mRNA levels compared to Fl/Fl mice. While the significance of these opposing changes is not clear, we propose that these distinct responses in mRNA levels of clock (*Bmal1*, *Clock*, and *Nr1d1*/REV-ERBα) and clock-controlled (*Nfil3*/E4BP4, *Noct*, and *Dbp*) genes most likely underpins many of the intriguing outcomes in lipid metabolism we observed in livers of LKO mice exposed to alcohol.

Only a handful of studies investigating ALD have considered the temporal nature of lipid metabolism and the molecular clock (Kudo et al., 2009; Filiano et al., 2013; Zhou et al., 2014; Zhang et al., 2018). Therefore, we first measured hepatic and plasma TG levels over a 24 h period. Expectedly, hepatic TG is higher in alcohol compared to control-fed mice over the course of the 24 h day. We also found a modest elevation in plasma TG in alcohol-fed Fl/Fl mice. Given that previous studies show dyslipidemia in mice with genetically altered clocks (Turek et al., 2005; Shimba et al., 2011; Bugge et al., 2012; Cho et al., 2012), we predicted that 24 h TG levels would be higher in livers from alcohol-fed LKO mice. Surprisingly, hepatic TG levels in alcohol-fed LKO mice were not dramatically elevated over levels measured in alcohol-fed Fl/Fl mice. We did however see higher and more variable levels of plasma TG in LKO mice fed alcohol compared to alcohol-fed Fl/Fl mice, supporting the hypothesis that the liver clock is important for consolidating hepatic TG export/uptake to particular times of day (Pan and Hussain, 2007; Pan et al., 2010). Interestingly, some of our findings differ from those reported in other studies. For example, alcohol-fed *Clock^Δ19/Δ19^* mutant mice have elevated hepatic, but unchanged plasma TG levels over the course of the day compared to wild-type mice fed alcohol (Kudo et al., 2009). Similarly, Zhang et al. (2018) reported higher TG in livers of LKO compared to wild-type mice when using the short-term “chronic + binge” alcohol model; however, hepatic TG was only reported at one time of day. Reasons for these disparate results likely include differences in the genetic mouse models of clock disruption, alcohol feeding protocol, and/or sex. Female mice were used in some of these studies (Kudo et al., 2009; Zhang et al., 2018). Female sex is associated with increased sensitivity to alcohol toxicity (Eagon 2010; Wagnerberger et al., 2013) and sex differences in clock gene rhythms are also known (Yang et al., 2009; Kuljis et al., 2013); thus, use of only male mice in our study may explain why we did not see higher TG content in livers of alcohol-fed LKO mice.

Accumulating evidence suggests that there may be time-of-day differences in susceptibility to alcohol-related liver injury. Voigt et al. (2017) showed diurnal variation in the integrity of the intestinal barrier and liver injury in mice subjected to alcohol “binges” (large one-time oral dose) given at different times of the day. Given this, we histologically examined livers to determine whether clock disruption increases markers of alcohol-related injury (e.g., steatosis, lobular inflammation, and hepatocyte ballooning) in a time-of-day dependent manner. We found that alcohol-fed LKO mice had the highest histopathology score with ballooned hepatocytes only detected in LKO mice. Mice with a disrupted liver clock also had a much higher percentage of hepatocytes containing lipid droplets as compared to Fl/Fl mice. Interestingly, alcohol induced more small vs. large droplet macrosteatosis, with the highest levels of small droplet macrosteatosis seen in livers of alcohol-fed LKO mice. The higher prevalence of small vs. large droplet macrosteatosis in livers of LKO mice likely explains the similarity in overall hepatic TG content in LKO and Fl/Fl mice, and highlights the importance of using both biochemical and histological measurements of steatosis in assessing fatty liver injury. Moreover, one limitation of only using H and E-stained liver sections in assessing steatosis is that lipid droplets are detected in the form of optically “empty” vacuoles in the tissue. Moving forward, it will be important to use neutral lipid (i.e., TG) staining reagents, such as BODIPY 493/503 to facilitate more rigorous visualization and quantification of lipid droplets in steatotic liver (Rasineni et al., 2014).

Notably, small droplet macrosteatosis is associated with reduced hepatic ATP content and poorer outcomes in liver transplant patients, suggesting a link to mitochondrial dysfunction (Ferri et al., 2019). Previously, we have shown that alcohol impairs mitochondrial bioenergetics (Young et al., 2006; King et al., 2014) and damages mtDNA (Venkatraman et al., 2004), with other studies reporting clock control of mitochondrial function (Peek et al., 2013; Jacobi et al., 2015). Thus, enhanced mitochondrial damage from the double “hit” of alcohol and clock disruption likely explains increased small droplet macrosteatosis in livers of alcohol-fed LKO mice.

We also observed time-of-day differences in steatosis. Small droplet macrosteatosis peaked in livers of alcohol-fed Fl/Fl mice during the early active period, whereas higher levels were seen in the inactive period for LKO mice. The inactive period of the day was also where we observed ballooned hepatocytes in livers of LKO mice, suggesting time-of-day dependent susceptibility to cell death. Ballooned hepatocytes are a hallmark feature of steatohepatitis, characterized by cell swelling, ubiquitinated protein accumulation, and intermediate cytoskeleton filament keratin 8/18 loss (Lackner et al., 2008; Mostafa et al., 2020). Interestingly, immune responsiveness and inflammation show day/night differences that are gated by the molecular clock (Keller et al., 2009; Nguyen et al., 2013; Curtis et al., 2015). Voigt et al. (2017) found diurnal variations in immune cell trafficking, with higher numbers of immune cells present in livers of alcohol-fed mice during the inactive period of the day. Taken together, these results support the concept that the degree of alcohol-related liver injury may be time-of-day dependent and/or influenced by the timing of alcohol consumption.

While transcriptomic studies indicate that mRNA levels of many lipid metabolism genes exhibit day/night differences (Adamovich et al., 2014; Aviram et al., 2016), the influence alcohol and liver clock disruption have on these diurnal rhythms in mRNA levels is not known. Therefore, we determined 24 h rhythms in mRNA levels for multiple lipid metabolism genes implicated in ALD. The first set of genes we examined were the canonical lipid metabolism transcription factors *Srebf1*/SREBP-1c, *Mlxip*l/ChREBP, *Nr1h3*/LXRα, *Nr1h2*/LXRβ, and *Ppara*. Arrhythmic mRNA levels were found in livers of LKO mice, suggesting clock-regulation of these transcription factors. We observed arrhythmic mRNA levels of *Srebf1*/SREBP-1c in livers of alcohol-fed Fl/Fl mice, leading to disrupted diurnal rhythms in mRNA levels for the SREBP-1c target genes *Acaca*/ACC1 and *Fasn*. Gaucher et al. (2019) also found that *Srebf1*/SREBP-1c and targets were repressed by 6 weeks of alcohol consumption. While it might be predicted that *Srebf1*/SREBP-1c mRNA levels would be elevated in alcohol-induced fatty liver, You et al. (2002) reported that alcohol increases *de novo* lipogenesis post-transcriptionally by increasing translocation of the mature form of SREBP-1c protein into the nucleus. Attempts to assess time-of-day rhythms in SREBP-1c protein translocation in the current study were unsuccessful due to poor antibody specificity (data not shown). Like *Srebf1*/SREBP-1c, *Nr1h2*/LXRβ was also rhythmic in livers of control-fed Fl/Fl, but arrhythmic in livers of alcohol-fed Fl/Fl mice. We propose that the alcohol-mediated loss of rhythmic *Nr1h2*/LXRβ mRNA levels drive arrhythmic *Srebf1*/SREBP-1c, a known target of LXRα/β (Repa et al., 2000). Significant main effects of genotype and diet were observed for both *Sreb1f*/SREBP-1c and *Nr1h2*/LXRβ in livers of LKO compared to Fl/Fl mice, further suggesting crosstalk between these two main lipid metabolism transcription factors in alcohol-exposed liver.

We also assessed diurnal rhythms in mRNA levels of *Ppara*, a transcriptional regulator of genes involved in multiple energy metabolism pathways, including FA transport and oxidation (Pawlak et al., 2015). Interestingly, *Ppara* mRNA levels were rhythmic in livers of alcohol-fed Fl/Fl mice, but arrhythmic in livers of control-fed Fl/Fl mice. With this said, the overall mRNA levels of *Ppara* were significantly lower in livers of alcohol-fed Fl/Fl mice and LKO mice, suggesting alcohol-related suppression of the hepatic FAO pathway. This finding is supported by other studies showing that acetaldehyde, the oxidative product of alcohol metabolism, inhibits DNA binding and transcriptional activity of PPARα and decreases FAO (Galli et al., 2001). Zhang et al. (2018) showed that treatment with fenofibrate, a synthetic PPARα ligand, and overexpression of ChREBP alleviate alcohol-related steatosis by activating FAO. Here, we found alcohol and clock-related decreases in the mesor of *Mlxpil*/ChREBP in the liver. Taken together, we propose that the combined effects of alcohol and clock disruption on *Srebf1*/SREBP-1c, *Nr1h2*/LXRβ, and/or *Ppara* rhythms impair oscillations of target FA and TG metabolism genes, contributing to steatosis.

A key regulatory node for partitioning lipids is the intersection of reactions involved in FA synthesis and oxidation, namely ACC1, ACC2, and MCD. *Acaca*/ACC1 catalyzes the generation of malonyl-CoA, which serves as the substrate for FA synthesis (McGarry et al., 1978b). In contrast, malonyl-CoA synthesized by *Acacb*/ACC2 functions as an allosteric inhibitor of CPT1a by inhibiting FA transport into mitochondria for FAO (McGarry et al., 1978a). These complementary enzymes help ensure that FA synthesis and oxidation do not occur simultaneously. In keeping with this, we found that *Acaca*/ACC1 and *Acacb*/ACC2 rhythms have different peaks in livers of control-fed Fl/Fl mice. *Acaca*/ACC1 peaks during the inactive period, whereas *Acacb*/ACC2 peaks during the active period of the day. We also observed that alcohol significantly dampened rhythms of both genes. At the protein level, AMPK phosphorylates and inactivates ACC, blocking malonyl-CoA production (Munday et al., 1988; Fullerton et al., 2013). Importantly, alcohol inhibits AMPK (Garcia-Villafranca et al., 2008), which is predicted to increase ACC activity and malonyl-CoA production, resulting in increased FA synthesis and decreased FAO – two potential mechanisms of steatosis. Therefore, we chose to assess both total and phosphorylated ACC1 and ACC2. Unfortunately, the results from these studies were equivocal. First, a p-ACC2 antibody is not available, thus, we had to use an antibody that detects both p-ACC1 and p-ACC2. Second, because these two proteins have high molecular weights of similar size (e.g., ACC1 = 265 kDa and ACC2 = 280 kDa), we were not able to achieve sufficient separation of proteins to analyze each band separately for densitometry. Therefore, bands were analyzed together. A significant main effect of diet was found for p-ACC1/2 with significantly lower levels in livers of alcohol-fed LKO mice. Accordingly, we predict that ACC1/2 activity and malonyl-CoA levels would be increased. We also assessed mRNA and proteins levels of *Mlycd*/MCD, the enzyme responsible for decarboxylation of malonyl-CoA back to acetyl-CoA (Saggerson, 2008). *Mlycd*/MCD mRNA levels were rhythmic in livers of control-fed Fl/Fl mice, with alcohol inducing a significant 3.6 h phase delay in the peak of the rhythm. MCD protein levels were stable throughout the day; however, there was a significant main effect of genotype with 50% less MCD protein in livers of LKO mice compared to Fl/Fl mice. Thus, these results predict higher levels of malonyl-CoA in livers of alcohol-fed LKO mice that will increase FA synthesis and/or decrease FAO oxidation, leading to steatosis. Previous studies show that *Mlycd*/MCD is a PPARα target, as decreased MCD mRNA levels/activity and FAO are present in hearts of PPARα knockout mice (Campbell et al., 2002). Future studies are planned to determine whether temporal dysregulation in a PPARα-ACC1/2-MCD-malonyl-CoA axis promotes alcohol-induced steatosis, especially in circadian clock disrupted livers.

Adamovich et al. (2014) reported that the enzymes involved in TG synthesis and breakdown exhibit diurnal variations in mRNA levels. Our results are consistent with these findings, showing significant diurnal rhythms in mRNA levels for *Gpat1*, *Agpat1/2*, *Lpin1/2*, *Dgat2*, and *Pnpla2*/3 in livers of control-fed Fl/Fl mice, with changed rhythms in livers of LKO mice. We predicted alcohol feeding would increase mRNA levels of TG synthesis genes; however, we largely observed dampening of rhythms. Importantly, alcohol significantly decreased the mesor and amplitude of *Pnpla2*/ATGL and *Pnpla3* rhythms. *Pnpla2*/ATGL is regarded as the rate-limiting enzyme in TG breakdown where it translocates to the surface of lipid droplets, binds *Abdh5*/CGI-58, and catalyzes the breakdown of TG to DG. Similarly, *Pnpla3* is a TG hydrolase with LPA transacylase activity (Jenkins et al., 2004; Lake et al., 2005; He et al., 2010); however, the physiological relevance of *Pnpla3* in TG metabolism remains ambiguous as targeted deletion of *Pnpla3* does not impair TG hydrolysis or promote steatosis (Basantani et al., 2011). In contrast, a missense genetic variant in PNPLA3 (I148M) in humans is strongly associated with fatty liver disease (Romeo et al., 2008) by reduced hydrolase activity (Huang et al., 2011) and disruption of *Pnpla2*/ATGL function on the lipid droplet surface (BasuRay et al., 2019; Wang et al., 2019). Interestingly, *Pnpla3* mRNA levels were dramatically reduced and arrhythmic in livers of LKO mice, which is similar to findings using *Per1/2* double knockout mice (Adamovich et al., 2014), suggesting a strong role of the clock in regulating *Pnpla3* mRNA levels in the liver.

The lipid droplet structure is maintained by PLIN1–5 with PLIN2 being the most abundant PLIN in liver (Itabe et al., 2017). Briefly, phosphorylation of PLIN2 by protein kinase A recruits PNPLA2/ATGL and PNPLA3 to the lipid droplet surface and initiates TG hydrolysis to DG, with subsequent breakdown reactions catalyzed by *Lipe*/HSL and *Mgll*, resulting in release of glycerol and FA (Zechner et al., 2009). We observed significant diurnal rhythms for *Plin2*, *Lipe*, and *Mgll*, but not *Plin5* and *Abdh5*/CGI-58 in livers of control-fed Fl/Fl mice. A role for PLIN2 in ALD is supported by studies showing alcohol-related increases in PLIN2 mRNA and protein in liver (Carr et al., 2013; Rasineni et al., 2014) and *Plin2* knockout mice are protected from ALD (Carr et al., 2014). Here, we found lower and higher levels of *Plin2* at ZT 3 and ZT 7, respectively, in livers of alcohol-fed Fl/Fl mice compared to control-fed Fl/Fl mice, suggesting alcohol-related dysregulation in mRNA levels of *Plin2*. This finding also highlights the importance of assessing 24 h rhythms in mRNA levels vs. only one time point during the day as a completely different alcohol-related response in mRNA levels were observed in only a 4 h time window for *Plin2*. PLIN5 is proposed to facilitate FA uptake from lipid droplets to mitochondria for FAO (Wang et al., 2011). Live cell imaging shows reduced contact between mitochondria and lipid droplets and decreased FAO in hepatocytes from *Plin5* knockout mice (Keenan et al., 2019). Here, *Plin5* mRNA levels were arrhythmic in livers of Fl/Fl mice, but rhythmic in livers of LKO mice with peak phase occurring at ZT 14. This finding suggests that *Plin5* mRNA levels may entrain to the feeding/fasting cycle in the absence of a functional liver clock; however, additional studies are needed to verify this finding. In contrast, *Plin2*, *Abdh5*/CGI-58, *Lipe*/HSL, and *Mgll* were all arrhythmic in livers of LKO mice. These novel findings suggest an important role of the circadian clock in maintaining temporal control of lipid droplet dynamics in hepatocytes.

In addition to lipolysis, lipophagy significantly contributes to lipid droplet catabolism by selectively targeting lipid droplets to lysosomes for degradation (Singh et al., 2009). In fact, several of the lipid droplet components we measured in this current study also participate in lipophagy. For example, PLIN2 functions as a substrate for chaperone-mediated lipophagy by recruiting *Pnpla2*/ATGL and autophagy-related proteins to the lipid droplet (Kaushik and Cuervo, 2015). *Pnpla2*/ATGL-mediated signaling through SIRT1 also stimulates lipophagy in hepatocytes (Sathyanarayan et al., 2017). It is also well-known that chronic alcohol impairs lipophagy, which along with lipolysis inhibition promotes developments of steatosis (Schulze et al., 2017; Chao and Ding, 2019). Interestingly, new work shows differential morphology in lipid droplet size when inhibiting lipolysis or lipophagy in hepatocytes (Schott et al., 2019). Specifically, inhibition of *Pnpla2*/ATGL with atglistatin results in accumulation of large lipid droplets, whereas inhibition of the lysosomal lipophagy pathway with chloroquine results in accumulation of small lipid droplets enriched in autophagic (LC3) and lysosomal (LAMP2A) markers in hepatocytes (Schott et al., 2019). While beyond the scope of this current paper, it will be important that future studies determine if impairment in lipophagy underpins increased small droplet macrosteatosis in livers of mice with a disrupted liver clock.

Previous studies have reported that alcohol alters the profile of multiple lipid species in the liver, including glycerolipids, glycerophospholipids, sphingolipids, and ceramides (see review Clugston et al., 2017). Important for this current study is work showing that the majority of oscillating lipids in the liver are TG species (Adamovich et al., 2014). Based on this, we used an MS/MS^ALL^-based lipidomics approach (Gajenthra Kumar et al., 2018) to provide novel mechanistic insights into how alcohol and clock disruption affect TG metabolism and steatosis. Specifically, we defined two characteristics of the hepatic TG pool – the overall TG FA saturation state and TG FA composition at ZT 3 and ZT 15 as these times generally corresponded to the nadir and peak of total TG content in livers of alcohol-fed Fl/Fl mice. First, we found lower and higher percentages of TG SFA and TG DUFA, respectively, in livers of alcohol-fed Fl/Fl mice. These findings coincide with data from other labs reporting decreases in SFA and increases in MUFA and PUFAs in livers of alcohol-fed mice and rats, representing a shift toward FA unsaturation (Clugston et al., 2011; Fernando et al., 2011). Accordingly, livers from alcohol-fed LKO mice also had higher TG MUFA and TG DUFA content, but lower TG PUFA content, especially at ZT 3. It should also be pointed out that FA saturation is likely linked to the composition of the Lieber-DeCarli diet that is high in unsaturated FA (Lieber et al., 1989).

Regarding FA composition, we found that alcohol and liver clock disruption differentially affect the FA profile of the hepatic TG pool in a time-of-day dependent manner. The most abundant FA detected in the TG pool were palmitic acid (16:0), oleic acid (18:1), and linoleic acid (18:2). Interestingly, palmitic acid (16:0) was significantly decreased in livers of alcohol-fed LKO mice at both time points compared to Fl/Fl mice, which likely correlates with much lower FASN levels in livers of LKO mice. Interestingly, Chakravarthy et al. (2005) found that a lack of FASN (by liver-specific FASN KO) did not protect against the development of hepatic steatosis, but rather increased steatosis due to reduced mitochondrial FAO in response to increased malonyl-CoA levels. As mentioned earlier, malonyl-CoA inhibits CPT1, the enzyme responsible for entry of fatty acyl-CoA into mitochondria for oxidation. Similarly, they hypothesized that newly FASN-synthesized palmitic acid functions as an endogenous ligand for PPARα, which would have the effect of increasing FAO in mitochondria. In support of this hypothesis, other studies have reported that saturated FA like palmitic acid bind to PPARα with affinities similar to those of unsaturated fatty acids (Kliewer et al., 1997; Xu et al., 1999). Taken together, these findings indicate that an alcohol-dependent decrease in MCD, FASN, and FASN-derived palmitic acid likely contributes to hepatic steatosis by decreasing mitochondrial FAO in the liver.

In contrast, we observed increased linoleic acid (18:2) in livers of alcohol-fed LKO mice at ZT 3. Other studies have also shown alcohol-related increases in 18 carbon FA of varying saturation in the total liver lipid pool (Puri et al., 2016). Importantly, Kirpich and colleagues have found that linoleic acid (18:2) increases alcohol-related liver injury in mice through production of oxidized linoleic acid metabolites that activate pro-inflammatory macrophages (Warner et al., 2017). Thus, it is possible that this mechanism contributed to increased injury in LKO mice. We also found large increases in the lower abundant 20 carbon FA, arachidic acid (20:0), eicosanoid acid (20:1), and eicosadienoic acid (20:2) in livers of alcohol-fed LKO at ZT 15, indicating a time-dependent shift in accumulation. We also observed interesting effects on arachidonic acid (20:4), an important precursor for synthesis of eicosanoids and other biologically active metabolites involved in cellular signaling and inflammatory response (Calder, 2012). Here, we found significantly lower levels of arachidonic acid (20:4) in livers of alcohol-fed mice, specifically in LKO mice at ZT 3, which is consistent with other studies in mice (Bradford et al., 2008; Loftus et al., 2011). However, alcohol increases arachidonic acid (20:4) in livers of alcohol-fed rats, suggesting species differences in FA turnover (Loftus et al., 2011). We also observed the highest levels of docosenoic acid (22:1) in livers of LKO mice that were further elevated at ZT 15. Finally, we observed a trend toward increased docosahexanoic acid (DHA, 22:6) in livers of alcohol-fed mice. Other studies have reported alcohol-related increases in DHA (Clugston et al., 2011; Puri et al., 2016), an important omega-3 PUFA with anti-inflammatory properties (Calder, 2012).

Based on these lipidomics finding, we would have predicted to have found a more pronounced disruption in the time-of-day patterns in mRNA levels of FA biosynthesis genes *Scd1*, *Elovl5/6*, and *Fads1/2*. We did however observe arrhythmic mRNA levels for all genes in livers of LKO mice, and alcohol-related alterations in *Elovl6* and *Fads2* rhythms in livers of Fl/Fl mice. These changes may have contributed to differences in TG FA composition between genotypes. Moving forward, future studies should incorporate assessments at the protein level as posttranslational modifications like phosphorylation and acetylation are important determinants of FA metabolism enzyme activity. Additionally, assessing TG FA composition over the entire 24 h period will provide a more complete picture of the dynamic nature of the hepatic lipidome.

## Concluding Remarks

In conclusion, the current study provides several novel observations regarding alcohol-related changes in lipid metabolism that may be of relevance in ALD. These studies reveal that liver clock disruption increases small droplet macrosteatosis in livers of alcohol-fed mice and significantly disrupts 24 h rhythms in mRNA levels of multiple clock and lipid metabolism genes in the liver. Importantly, we also found that alcohol, liver clock disruption, or both conditions in combination significantly remodeled the FA profile of the hepatic TG pool lipidome. Taken together, these novel findings highlight the importance of a functional liver molecular clock in maintaining hepatic lipid homeostasis, and suggest that circadian clock disruption may be an important risk factor in the pathogenesis of ALD.

## Data Availability Statement

The raw data supporting the conclusions of this article will be made available by the authors, without undue reservation.

## Ethics Statement

The animal study was reviewed and approved by UAB Institutional Animal Care and Use Committee.

## Author Contributions

JV, UU, TS, and KA performed research. CP and SD performed histology scoring. JV and PB performed mass spectrometry. JV, KG, and SB performed statistical analyses. KG and SB designed study. JV and SB wrote manuscript with co-author input and review. All authors contributed to the article and approved the submitted version.

### Conflict of Interest

PB was employed by the Avanti Polar Lipids, Inc (USA).

The remaining authors declare that the research was conducted in the absence of any commercial or financial relationships that could be construed as a potential conflict of interest.
