# Supplementary material for: Alcohol and Liver Clock Disruption Increase Small Droplet Macrosteatosis, Alter Lipid Metabolism and Clock Gene mRNA Rhythms, and Remodel the Triglyceride Lipidome in Mouse Liver
Source: Front Physiol. 2020 Sep 7;11:1048. doi: 10.3389/fphys.2020.01048 (PMC7504911; doi:10.3389/fphys.2020.01048)
Supplement: Supplementary file 1 [file Data_Sheet_1.pdf]

Supplemental Table 1. Cosinor analysis of clock gene mRNA levels in livers of control and alcohol-fed *Bmal1* LKO and control genotype mice.

			Rhythmicity		Cosinor Parameters					
	Genotype	Diet	R^2	p-value R^2	Mesor	p-value t-test	Amplitude	p-value t-test	Acrophase	p-value t-test
Bmal1	Flox	Con	**0.52**	2.0E-06	**14.89**		**14.26**		22.2	
		ETOH	**0.32**	0.003	**9.69**	0.02	**6.98**	0.02	22.7	-
	LKO	Con	**0.59**	2.0E-06	2.22		1.68		22.8	
		ETOH	**0.52**	1.3E-05	1.77	-	1.38	-	23.7	-
	Flox	Con	**0.52**	2.0E-06	**14.89**		**14.26**		22.2	
	LKO	Con	**0.59**	2.0E-06	**2.22**	3.1E-8	**1.68**	1.20E-5	22.8	-
	Flox	ETOH	**0.32**	0.003	**9.69**		**6.98**		22.7	
	LKO	ETOH	**0.52**	1.3E-05	**1.77**	5.6E-7	**1.38**	0.005	23.7	-
Clock	Flox	Con	**0.29**	0.004	1.46		0.52		22.3	
		ETOH	0.05	0.51	-	-	-	-	-	-
	LKO	Con	0.01	0.88	-		-		-	
		ETOH	0.10	0.23	-	-	-	-	-	-
Rev-erba	Flox	Con	**0.79**	7.6E-08	11.54		14.73		**7.5**	
		ETOH	**0.82**	1.6E-08	9.71	-	10.49	-	**4.7**	0.0004
	LKO	Con	**0.90**	1.6E-11	3.03		**2.91**		10.8	
		ETOH	**0.77**	6.7E-07	2.56	-	**2.05**	0.04	10.8	-
	Flox	Con	**0.79**	7.6E-08	**11.54**		**14.73**		**7.5**	
	LKO	Con	**0.90**	1.6E-11	**3.03**	1.3E-6	**2.91**	1.86E-6	**10.8**	4.50E-6
	Flox	ETOH	**0.82**	1.6E-08	**9.71**		**10.49**		**4.7**	
	LKO	ETOH	**0.77**	6.7E-07	**2.56**	2.4E-8	**2.05**	5.65E-7	**10.8**	1.63E-5
Per2	Flox	Con	**0.68**	3.5E-09	3.36		2.41		13.1	
		ETOH	**0.56**	3.0E-06	2.95	-	1.69	-	12.1	-
	LKO	Con	**0.50**	4.0E-05	3.79		1.81		14.9	
		ETOH	0.15	0.09	-	-	-	-	-	-
	Flox	Con	**0.68**	3.5E-09	3.36		2.41		**13.1**	
	LKO	Con	**0.50**	4.0E-05	3.79	-	1.81	-	**14.9**	0.04
	Flox	ETOH	**0.56**	3.0E-06	2.95		1.69		12.1	
	LKO	ETOH	0.15	0.09	-	-	-	-	-	-
Cry1	Flox	Con	**0.62**	1.09E-07	2.19		1.66		19.5	
		ETOH	**0.63**	5.8E-07	1.81	-	1.28	-	20.3	-
	LKO	Con	0.11	0.16	-		-		-	
		ETOH	0.07	0.32	-	-	-	-	-	-
Csnk1d	Flox	Con	0.01	0.82	-		-		-	
		ETOH	0.02	0.73	-	-	-	-	-	-
	LKO	Con	0.04	0.54	-		-		-	
		ETOH	0.09	0.24	-	-	-	-	-	-
Csnk1e	Flox	Con	0.15	0.07	-		-		-	
		ETOH	0.18	0.61	-	-	-	-	-	-
	LKO	Con	0.11	0.85	-		-		-	
		ETOH	0.06	0.43	-	-	-	-	-	-
Noct	Flox	Con	**0.57**	7.0E-07	2.96		2.19		11.4	
		ETOH	0.12	0.15	-	-	-	-	-	-
	LKO	Con	**0.33**	0.002	1.89		0.91		12.5	
		ETOH	0.09	0.24	-	-	-	-	-	-
	Flox	Con	**0.57**	7.0E-07	**2.96**		**2.19**		11.4	
	LKO	Con	**0.33**	0.002	**1.89**	0.001	**0.91**	0.007	12.5	-
Dbp	Flox	Con	**0.59**	2.0E-06	**30.74**		**37.44**		8.6	
		ETOH	**0.71**	2.4E-07	**16.28**	0.003	**17.28**	0.003	7.3	-
	LKO	Con	**0.54**	2.8E-05	7.70		6.17		8.1	
		ETOH	0.10	0.20	-	-	-	-	-	-
	Flox	Con	**0.59**	2.0E-06	**30.74**		**37.44**		8.6	
	LKO	Con	**0.54**	2.8E-05	**7.70**	8.2E-6	**6.17**	1.24E-5	8.1	-
	Flox	ETOH	**0.71**	2.4E-07	16.28		17.28		7.3	
	LKO	ETOH	0.10	0.20	**-**	-	**-**	-	-	-

***Nfil3*/E4BP4**	Flox	Con	**0.22**	0.03	1.87		1.02		22.7	
		ETOH	0.08	0.08	-	-	-	-	-	-
	LKO	Con	0.02	0.56	-		-		-	
		ETOH	0.01	0.78	-	-	-	-	-	-

For rhythmicity, R^2 values in bold correspond to results with significant p-value ($p<0.05$). Cosinor parameters in bold correspond to results with significant p-value ($p<0.05$) by Student's t-test.

Supplemental Table 2. Two-Factor Analysis of Variance (ANOVA) for clock gene mRNA levels.

	**Genotype**		**Diet**		**Genotype X Diet**	
	**F**	**p-value**	**F**	**p-value**	**F**	**p-value**
Bmal1	**50.81**	5.83E-11	**4.24**	0.041	3.78	0.054
Clock	**17.65**	5.10E-5	**4.85**	0.030	0.02	-
Rev-erba	**24.78**	2.00E-6	0.95	-	0.51	-
Per2	**8.82**	0.004	0.49	-	0.03	-
Cry1	**313.65**	1.85E-36	**6.39**	0.013	2.43	-
Csnk1d	**29.63**	2.34E-7	0.23	-	0.29	-
Csnk1e	**34.30**	3.51E-8	2.65	-	2.60	-
Noct	**4.63**	0.033	3.43	0.066	0.39	-
Dbp	**11.61**	0.001	**5.29**	0.023	3.52	0.063
Nfil3	2.14	-	**7.13**	0.009	2.93	0.090

F-values in bold font correspond with results showing significant p-values ($p < 0.05$) by two-factor ANOVA. Gray font p-values just missed statistical significance. A dash indicates $p > 0.10$

Supplemental Table 3. Cosinor analysis for mRNA levels of lipid metabolism transcription factors in livers of control and alcohol-fed *Bmal1* LKO and Fl/Fl mice.

			Rhythmicity		Cosinor Parameters					
	**Genotype**	**Diet**	**R^2**	**p-value R^2**	**Mesor**	**p-value t-test**	**Amplitude**	**p-value t-test**	**Acrophase**	**p-value t-test**
***Srebf1/* SREBP-1c**	Flox	Con	**0.19**	0.03	1.31		0.20		5.4	
		ETOH	0.07	0.4	-	-	-	-	-	-
	LKO	Con	0.07	0.3	-		-		-	
		ETOH	0.02	0.8	-	-	-	-	-	-
***Mlxipl/* ChREBP**	Flox	Con	0.06	0.4	-		-		-	
		ETOH	0.14	0.1	-	-	-	-	-	-
	LKO	Con	0.03	0.6	-		-		-	
		ETOH	0.04	0.6	-	-	-	-	-	-
***Nr1h3/* LXRα**	Flox	Con	0.0003	1.0	-		-		-	
		ETOH	0.05	0.4	-	-	-	-	-	-
	LKO	Con	0.04	0.5	-		-		-	
		ETOH	0.03	0.6	-	-	-	-	-	-
***Nr1h2/* LXRβ**	Flox	Con	**0.28**	0.004	1.23		0.23		4.5	
		ETOH	0.05	0.5	-	-	-	-	-	-
	LKO	Con	0.07	0.4	-		-		-	
		ETOH	0.05	0.5	-	-	-	-	-	-
Pparα	Flox	Con	0.03	0.5	-		-		-	
		ETOH	**0.34**	0.01	1.06	-	0.45	-	7.5	-
	LKO	Con	0.10	0.2	-		-		-	
		ETOH	0.04	0.2	-	-	-	-	-	-

For rhythmicity, R^2 values in bold correspond to results with significant p-value ($p<0.05$).

Supplemental Table 4. Two-Factor Analysis of Variance (ANOVA) for lipid metabolism gene mRNA levels.

	Genotype		Diet		Genotype X Diet	
	**F**	**p-value**	**F**	**p-value**	**F**	**p-value**
Abdh5	**60.60**	1.58E-12	**12.78**	4.86E-4	2.89	0.091
Acaca	**43.14**	1.01E-9	0.99	-	2.94	0.089
Acacb	**16.38**	8.90E-5	**14.97**	1.70E-4	**5.51**	0.020
Agpat1	**6.55**	0.012	2.82	0.096	0.36	-
Agpat2	**16.63**	8.20E-5	1.84	-	1.56	-
Chrebp	**32.88**	6.07E-8	0.76	-	3.79	0.054
Cpt1a	2.49	-	2.99	0.086	0.50	-
Dgat2	0.95	-	0.48	-	0.25	-
Elovl5	1.72	-	0.01	-	0.07	-
Elovl6	**5.02**	0.027	**15.08**	1.65E-4	0.59	-
Fads1	**14.38**	2.34E-4	1.82	-	0.03	-
Fads2	**9.40**	0.003	0.20	-	0.29	-
Fasn	0.47	-	1.52	-	**10.30**	0.002
Gpat1	2.51	-	2.00	-	0.51	-
Lipe	**8.57**	0.004	**7.62**	0.007	1.65	-
Lpin1	0.27	-	**7.32**	0.008	1.41	-
Lpin2	1.97	-	**3.92**	0.050	1.67	-
Mgll	**24.93**	2.00E-6	0.05	-	0.36	-
Mlycd	**21.74**	7.00E-6	**27.40**	6.12E-7	3.73	0.056
Nr1h2	**14.62**	1.99E-4	**8.79**	0.004	0.576	-
Nr1h3	3.24	0.074	**4.74**	0.031	0.008	-
Plin2	0.20	-	**9.69**	0.002	**4.98**	0.027
Plin5	**6.23**	0.014	**5.69**	0.018	2.95	0.088
Pnpla2	0.007	-	**13.61**	3.27E-4	0.44	-
Pnpla3	**11.68**	0.001	**8.19**	0.005	**8.28**	0.005
Ppara	**12.34**	0.001	**9.67**	0.002	0.004	-
Scd1	**34.75**	2.82E-8	0.017	-	0.107	-
Srebpf1	**24.09**	3.00E-6	**13.70**	3.10E-4	0.203	-

F-values in bold font correspond with results showing significant p-values ($p < 0.05$) by two-factor ANOVA. Gray font p-values just missed statistical significance. A dash indicates $p > 0.10$.

Supplemental Table 5. Cosinor analysis of fatty acid (FA) metabolism gene mRNA levels in livers of control and alcohol-fed *Bmal1* LKO and control genotype mice.

			Rhythmicity		Cosinor Parameters					
	Genotype	Diet	R^2	p-value R^2	Mesor	p-value t-test	Amplitude	p-value t-test	Acrophase	p-value t-test
***Acaca*/ ACC1**	Flox	Con	**0.36**	0.001	1.46		0.42		9.9	
		ETOH	0.14	0.1	-	-	-	-	-	-
	LKO	Con	0.07	0.4	-		-		-	
		ETOH	0.13	0.1	-	-	-	-	-	-
***Acacb*/ ACC2**	Flox	Con	**0.41**	1.7E-4	**1.48**		0.56		15.8	
		ETOH	**0.35**	0.001	**0.92**	2.0E-6	0.31	-	16.6	-
	LKO	Con	**0.22**	0.03	0.95		0.28		23.1	
		ETOH	0.07	0.3	-	-	-	-	-	-
	Flox	Con	**0.41**	1.7E-4	**1.48**		0.56		**15.8**	
	LKO	Con	**0.22**	0.03	**0.95**	2.6E-5	0.28	-	**23.1**	2.2E-5
***Mlycd*/ MCD**	Flox	Con	**0.51**	4.0E-6	**1.47**		0.33		**3.9**	
		ETOH	**0.18**	0.04	**1.31**	0.03	0.24	-	**7.5**	0.03
	LKO	Con	**0.40**	0.001	1.89		0.27		9.8	
		ETOH	0.09	0.20	-	-	-	-	-	-
	Flox	Con	**0.51**	4.0E-6	**1.47**		0.33		**3.9**	
	LKO	Con	**0.40**	0.001	**1.89**	4.7E-8	0.27	-	**9.8**	3.2E-6
Cpt1a	Flox	Con	**0.22**	0.01	1.45		0.32		3.3	
		ETOH	**0.40**	2.1E-4	1.39	-	0.35	-	4.2	-
	LKO	Con	0.04	0.5	-		-		-	
		ETOH	**0.24**	0.01	1.45	-	0.42	-	1.2	-
	Flox	ETOH	**0.40**	2.1E-4	1.39		0.35		4.2	
	LKO	ETOH	**0.24**	0.01	1.45	-	0.42	-	1.2	-
Fasn	Flox	Con	**0.20**	0.02	1.46		0.47		14.0	
		ETOH	**0.22**	0.02	1.21	-	0.40	-	15.6	-
	LKO	Con	0.16	0.06	-		-		-	
		ETOH	0.03	0.6	-	-	-	-	-	-
Scd1	Flox	Con	0.01	0.8	-		-		-	
		ETOH	0.06	0.4	-	-	-	-	-	-
	LKO	Con	0.04	0.6	-		-		-	
		ETOH	0.003	1.0	-	-	-	-	-	-
Elovl5	Flox	Con	**0.22**	0.04	1.28		0.37		23.6	
		ETOH	**0.29**	0.007	1.31	-	0.66	-	23.6	-
	LKO	Con	0.14	0.1	-		-		-	
		ETOH	0.10	0.2	-	-	-	-	-	-
Elovl6	Flox	Con	**0.30**	0.005	1.79		0.78		12.5	
		ETOH	0.08	0.3	-	-	-	-	-	-
	LKO	Con	0.007	0.9	-		-		-	
		ETOH	0.09	0.2	-	-	-	-	-	-
Fads1	Flox	Con	0.08	0.1	-		-		-	
		ETOH	0.05	0.4	-	-	-	-	-	-
	LKO	Con	0.08	0.9	-		-		-	
		ETOH	0.07	0.9	-	-	-	-	-	-
Fads2	Flox	Con	**0.23**	0.02	1.58		0.59		10.0	
		ETOH	0.13	0.1	-	-	-	-	-	-
	LKO	Con	0.03	0.6	-		-		-	
		ETOH	0.01	0.8	-	-	-	-	-	-

For rhythmicity, R^2 values in bold correspond to results with significant p-value ($p<0.05$). Cosinor parameters in bold correspond to results with significant p-value ($p<0.05$) by Student's t-test

Supplemental Table 6. Cosinor analysis of triglyceride metabolism gene mRNA levels in livers of control and alcohol-fed *Bmal1* LKO and control genotype mice.

			Rhythmicity		Cosinor Parameters					
	Genotype	Diet	R^2	p-value R^2	Mesor	p-value t-test	Amplitude	p-value t-test	Acrophase	p-value t-test
Gpat1	Flox	Con	**0.51**	0.04	1.16		0.15		14.0	
		ETOH	0.12	0.1	-	-	-	-	-	-
	LKO	Con	**0.31**	0.003	0.96		0.32		17.3	
		ETOH	0.03	0.7	-	-	-	-	-	-
	Flox	Con	**0.51**	0.04	**1.16**		**0.15**		14.0	
	LKO	Con	**0.31**	0.003	**0.96**	0.05	**0.32**	0.02	17.3	-
Agpat1	Flox	Con	**0.21**	0.04	1.04		0.13		10.4	
		ETOH	0.09	0.2	-	-	-	-	-	-
	LKO	Con	**0.39**	0.001	1.12		0.25		16.2	
		ETOH	**0.29**	0.007	1.06	-	0.19	-	15.6	-
	Flox	Con	**0.21**	0.04	1.04		0.13		**10.4**	
	LKO	Con	**0.39**	0.001	1.12	-	0.25	-	**16.2**	0.001
Agpat2	Flox	Con	**0.45**	0.04	1.36		0.23		19.1	
		ETOH	**0.63**	0.001	1.21	-	0.28	-	19.6	-
	LKO	Con	**0.52**	0.01	1.53		0.27		15.6	
		ETOH	0.23	0.5	-	-	-	-	-	-
	Flox	Con	**0.45**	0.04	1.36		0.23		**19.1**	
	LKO	Con	**0.52**	0.01	1.53	-	0.27	-	**15.6**	0.05
Lpin1	Flox	Con	**0.70**	4.3E-9	**3.37**		**3.24**		10.7	
		ETOH	**0.53**	1.2E-5	**1.84**	2.3E-05	**1.42**	3.0E-4	11.6	-
	LKO	Con	**0.38**	0.001	2.72		1.47		9.6	
		ETOH	0.15	0.1	-	-	-	-	-	-
	Flox	Con	**0.70**	4.3E-9	3.37		**3.24**		10.7	
	LKO	Con	**0.38**	0.001	2.72	-	**1.47**	0.002	9.6	-
Lpin2	Flox	Con	**0.50**	4.6E-5	1.79		0.81		10.7	
		ETOH	0.02	0.8	-	-	-	-	-	-
	LKO	Con	0.17	0.1	-		-		-	
		ETOH	0.12	0.2	-	-	-	-	-	-
Dgat2	Flox	Con	**0.49**	5.6E-4	1.36		**0.37**		10.0	
		ETOH	**0.20**	0.03	1.26	-	**0.19**	0.07	9.5	-
	LKO	Con	**0.27**	0.01	1.22		0.23		12.7	
		ETOH	0.16	0.08	-	-	-	-	-	-
	Flox	Con	**0.49**	5.6E-4	**1.36**		0.37		10.0	
	LKO	Con	**0.27**	0.01	**1.22**	0.05	0.23	-	12.7	-
***Pnpla2/* ATGL**	Flox	Con	**0.49**	8.0E-6	**2.22**		**0.88**		6.5	
		ETOH	**0.26**	0.008	**1.73**	0.001	**0.42**	0.03	5.2	-
	LKO	Con	0.08	0.3	-		-		-	
		ETOH	**0.22**	0.03	1.75	-	0.34	-	7.2	-
	Flox	ETOH	**0.26**	0.008	1.73		0.42		5.2	
	LKO	ETOH	**0.22**	0.03	1.75	-	0.34	-	7.2	-
Pnpla3	Flox	Con	**0.29**	0.002	**3.79**		**3.90**		15.7	
		ETOH	**0.25**	0.01	**0.88**	4.9E-4	**0.51**	0.003	17.5	-
	LKO	Con	0.13	0.1	-		-		-	
		ETOH	0.08	0.3	-	-	-	-	-	-

For rhythmicity, R^2 values in bold correspond to results with significant p-value ($p<0.05$). Cosinor parameters in bold correspond to results with significant p-value ($p<0.05$) by Student's t-test

Supplemental Table 7. Cosinor analysis of lipid droplet gene mRNA levels in livers of control and alcohol-fed *Bmal1* LKO and control genotype mice.

			Rhythmicity		Cosinor Parameters					
	**Genotype**	**Diet**	**R^2**	**p-value R^2**	**Mesor**	**p-value t-test**	**Amplitude**	**p-value t-test**	**Acrophase**	**p-value t-test**
Plin2	Flox	Con	**0.28**	0.004	1.50		0.47		1.0	
		ETOH	**0.17**	0.05	1.46	-	0.32	-	2.6	-
	LKO	Con	0.09	0.2	-		-		-	
		ETOH	0.01	0.8	-	-	-	-	-	-
Plin5	Flox	Con	0.09	0.2	-		-		-	
		ETOH	0.07	0.3	-	-	-	-	-	-
	LKO	Con	**0.21**	0.03	**1.69**		0.40		14.2	
		ETOH	**0.18**	0.05	**1.40**	0.03	0.28	-	14.3	-
***Abdh5/* CGI-58**	Flox	Con	0.05	0.4	-		-		-	
		ETOH	0.05	0.4	-	-	-	-	-	-
	LKO	Con	0.08	0.3	-		-		-	
		ETOH	0.05	0.4	-	-	-	-	-	-
Lipe	Flox	Con	**0.20**	0.03	1.19		0.19		9.7	
		ETOH	0.07	0.3	-	-	-	-	-	-
	LKO	Con	0.07	0.3	-		-		-	
		ETOH	0.02	0.7	-	-	-	-	-	-
Mgll	Flox	Con	**0.29**	0.008	1.91		1.43		3.6	
		ETOH	**0.24**	0.02	1.96	-	1.08	-	2.5	-
	LKO	Con	0.16	0.1	-		-		-	
		ETOH	0.02	0.5	-	-	-	-	-	-

For rhythmicity, R^2 values in bold correspond to results with significant p-value ($p<0.05$). Cosinor parameters in bold correspond to results with significant p-value ($p<0.05$) by Student's t-test.

Supplemental Table 8. TG species detected in livers and used to calculate % TG FA saturation (Fig 9) and % TG FA composition (Fig 10).

FA 12:0	FA 12:1	FA 14:0	FA 14:1	FA 16:0	FA 16:1	FA 18:1	FA 18:2	FA 20:0	FA 20:1	FA 20:2	FA 20:4	FA 22:1	FA 22:6
50:2	50:4	48:0	48:2	48:0	48:1	48:1	48:2	50:1	50:1	50:2	50:4	50:1	54:6
50:3	52:2	48:1	50:2	48:1	48:2	48:2	50:2	50:2	50:2	50:3	52:4	50:2	54:7
50:4	52:3	48:2	50:3	48:2	50:1	50:1	50:3	50:3	50:3	50:4	52:5	50:3	56:6
52:2	52:4	50:0	50:4	50:0	50:2	50:2	50:4	50:4	50:4	52:2	54:4	50:4	56:7
52:3	52:5	50:1	52:2	50:1	50:3	50:3	52:2	52:1	52:1	52:3	54:5	52:1	56:8
52:4	54:3	50:2	52:3	50:2	50:4	50:4	52:3	52:2	52:2	52:4	54:6	52:2	58:6
52:5	54:4	50:3	52:4	50:3	52:1	52:1	52:4	52:3	52:3	52:5	54:7	52:3	58:7
54:4	54:5	50:4	52:5	50:4	52:2	52:2	52:5	52:4	52:4	54:2	56:4	52:4	58:8
54:5	54:6	52:1	54:3	52:1	52:3	52:3	54:2	52:5	52:5	54:3	56:5	52:5	
	56:4	52:2	54:4	52:2	52:4	52:4	54:3	54:1	54:1	54:4	56:6	54:1	
	56:7	52:3	54:5	52:3	52:5	52:5	54:4	54:2	54:2	54:5	56:7	54:2	
	58:5	52:4	54:6	52:4	54:1	54:1	54:5	54:3	54:3	54:6	56:8	54:3	
	58:7	52:5	54:7	52:5	54:2	54:2	54:6	54:4	54:4	54:7	58:4	54:4	
	58:8	54:1	56:3	54:1	54:3	54:3	54:7	54:5	54:5	56:2	58:5	54:5	
		54:2	56:4	54:2	54:4	54:4	56:2	54:6	54:6	56:3	58:6	54:6	
		54:3	56:6	54:3	54:5	54:5	56:3	54:7	54:7	56:4	58:7	54:7	
		54:4	58:5	54:4	54:6	54:6	56:4	56:2	56:2	56:5	58:8	56:2	
		54:5	58:8	54:5	54:7	54:7	56:5	56:3	56:3	56:6		56:3	
		54:6		54:6	56:2	56:2	56:6	56:4	56:4	56:7		56:4	
		54:7		54:7	56:3	56:3	56:7	56:5	56:5	56:8		56:5	
		56:2		56:2	56:4	56:4	56:8	56:6	56:6	58:3		56:6	
		56:3		56:3	56:5	56:5	58:3	56:7	56:7	58:4		56:7	
		56:4		56:4	56:6	56:6	58:4	56:8	56:8	58:5		56:8	
		56:5		56:5	56:7	56:7	58:5	58:3	58:3	58:6		58:3	
		56:6		56:6	56:8	56:8	58:6	58:4	58:4	58:7		58:4	
		56:7		56:7	58:3	58:3	58:7	58:5	58:5	58:8		58:5	
		56:8		56:8	58:4	58:4	58:8	58:6	58:6			58:6	
		58:6		58:1	58:5	58:5		58:7	58:7			58:7	
		58:7		58:3	58:6	58:6		58:8	58:8			58:8	
				58:4	58:7	58:7							
				58:5	58:8	58:8							
				58:6									
				58:7									
				58:8									

Supplemental Table 9. Three-Factor Analysis of Variance (ANOVA) of liver triglyceride (TG) lipidomics.

	Genotype		Diet		Time		Genotype X Diet		Genotype X Time		Diet X Time		Genotype X Diet X Time	
	F	p-value	F	p-value	F	p-value	F	p-value	F	p-value	F	p-value	F	p-value
**SFA**	**14.9**	0.0008	**14.3**	0.0009	3.6	-	0.5	-	0.3	-	1.3	-	0.04	-
**MUFA**	3.4	-	**7.5**	0.01	**6.3**	0.02	2.5	-	0.3	-	3.6	-	0.1	-
**DUFA**	**12.9**	0.002	**5.5**	0.03	3.6	-	2.5	-	8E-04	-	5.5	-	2.4	-
**PUFA**	3E-03	-	**6.6**	0.02	0.2	-	2.6	-	5E-04	-	1.8	-	1.9	-
**12:0**	**23.6**	<0.0001	**10.2**	0.004	0.9	-	1.2	-	1.6	-	1.6	-	0.1	-
**12:1**	**13.2**	0.001	**5.0**	0.03	1.4	-	0.05	-	0.7	-	0.02	-	0.2	-
**14:0**	0.2	-	0.2	-	1.8	-	**7.6**	0.01	0.5	-	0.2	-	0.003	-
**14:1**	**8.3**	0.008	1.2	-	**7.3**	0.01	**23.6**	<0.0001	2.6	-	0.4	-	1.9	-
**16:0**	**14.7**	0.0008	**12.7**	0.002	3.8	-	0.4	-	0.4	-	1.1	-	0.03	-
**16:1**	**10.8**	0.003	0.03	-	2.3	-	0.2	-	0.3	-	1.1	-	0.1	-
**18:1**	3.9	-	**6.3**	0.02	**6.7**	0.02	2.4	-	0.4	-	2.2	-	0.1	-
**18:2**	**10.3**	0.004	**4.8**	0.04	4.0	-	2.4	-	8E-04	-	**6.1**	0.02	2.6	-
**20:0**	**123.3**	<0.0001	**17.4**	0.0005	**16.8**	0.0006	**29.2**	<0.0001	0.5	-	**11.0**	0.004	**4.9**	0.04
**20:1**	**33.8**	<0.0001	**6.2**	0.02	**8.9**	0.007	3.81	0.06	0.04	-	**14.6**	0.001	2.7	-
**20:2**	**15.8**	0.0006	0.9	-	**5.1**	0.03	0.4	-	0.01	-	**10.4**	0.004	**4.7**	0.04
**20:4**	0.5	-	**17.2**	0.0004	0.07	-	0.8	-	0.5	-	2.8	-	**4.3**	0.04
**22:1**	**169.6**	<0.0001	**44.1**	<0.0001	**23.1**	<0.0001	**38.6**	<0.0001	**11.2**	0.003	**12.3**	0.002	**5.8**	0.03
**22:6**	0.006	-	**5.2**	0.03	0.002	-	4.2	-	0.04	-	1.5	-	0.01	-

F-values in bold font correspond with results showing significant p-values ($p < 0.05$) by three-factor ANOVA. Gray font p-values just missed statistical significance. A dash indicates $p > 0.10$.

Supplemental Table 10. Results represent statistically significant p-values for select pair-wise comparisons between treatment groups.

Parameter	1 vs. 2	3 vs. 4	5 vs. 6	7 vs. 8	1 vs. 3	1 vs. 5	3 vs. 7	5 vs. 7	2 vs. 4	2 vs. 6	4 vs. 8	6 vs. 8
**20:0**	-	-	-	7.0E-5	0.032	-	-	-	3.1E-5	-	0.001	3.8E-7
**20:2**	-	-	-	0.047	-	-	-	-	-	-	0.027	0.064
**20:4**	-	0.003	-	-	-	-	-	-	-	-	-	-
**22:1**	-	0.007	-	2.0E-6	-	0.023	-	0.033	1.0E-4	-	5.7E-5	2.1E-8

Results in black font are p-values ($p < 0.05$) for pair-wise comparisons between treatment groups. Gray font p-values just missed statistical significance. A dash indicates $P > 0.10$
1 vs. 2 = Con Fl/Fl ZT3 vs. EtOH Fl/Fl ZT3
3 vs. 4 = Con LKO ZT3 vs. EtOH LKO ZT3
5 vs. 6 = Con Fl/Fl ZT15 vs. EtOH Fl/Fl ZT15
7 vs. 8 = Con LKO ZT15 vs. EtOH LKO ZT15
1 vs. 3 = Con Fl/Fl ZT 3 vs. Con LKO ZT 3
1 vs. 5 = Con Fl/Fl ZT 3 vs. Con Fl/Fl ZT 15
3 vs. 7 = Con LKO ZT 3 vs. Con LKO ZT 15
5 vs. 7 = Con Fl/Fl ZT 15 vs. Con LKO ZT 15
2 vs. 4 = EtOH Fl/Fl ZT 3 vs. EtOH LKO ZT 3
2 vs. 6 = EtOH Fl/Fl ZT 3 vs. EtOH Fl/FL ZT 15
4 vs. 8 = EtOH LKO ZT 3 vs. EtOH LKO ZT 15
6 vs. 8 = EtOH Fl/Fl ZT 15 vs. EtOH LKO ZT 15

Supplemental Table 11. Two-Factor Analysis of Variance (ANOVA) for Selected TG FAs

	Genotype		Diet		Genotype X Diet	
**Parameter**	**F**	**p-value**	**F**	**p-value**	**F**	**p-value**
**14:0**	0.20	-	0.23	-	**8.0**	0.009
**14:1**	**5.89**	0.02	0.72	-	**17.24**	3.0E-4

F-values in bold font correspond with results showing significant p-values ($p < 0.05$) by two-factor ANOVA. Gray font p-values just missed statistical significance. A dash indicates $p > 0.10$.

Supplemental Table 12. Two-Factor Analysis of Variance (ANOVA) for Selected TG FAs

	Diet		Time		Diet X Time	
**Parameter**	**F**	**p-value**	**F**	**p-value**	**F**	**p-value**
**18:2**	3.43	0.07	2.86	-	**4.33**	0.05
**20:1**	**5.21**	0.03	**5.35**	0.03	**6.81**	0.02

F-values in bold font correspond with results showing significant p-values ($p < 0.05$) by two-factor ANOVA. Gray font p-values just missed statistical significance. A dash indicates $p > 0.10$.

Supplemental Table 13. Primers used for RT-PCR measurements - Thermo-Fisher Cat# 4331182

Gene Name	Assay ID	Species
Abdh5	Mm00470734_m1	Mouse
Acaca	Mm01304277_m1	Mouse
Acacb	Mm01204671_m1	Mouse
Agpat1	Mm00479700_m1	Mouse
Agpat2	Mm00458880_m1	Mouse
Arntl	Mm00500226_m1	Mouse
Chrebp	Mm00498811_m1	Mouse
Cpt1a	Mm00550438_m1	Mouse
Cry2	Mm00546062_m1	Mouse
Csnk1d	Mm00503623_m1	Mouse
Csnk1e	Mm00443344_m1	Mouse
Dbp	Mm00497539_m1	Mouse
Dgat2	Mm00499536_m1	Mouse
Elovl5	Mm00506717_m1	Mouse
Elovl6	Mm00851223_s1	Mouse
Fads1	Mm00507605_m1	Mouse
Fads2	Mm00517221_m1	Mouse
Fasn	Mm00662319_m1	Mouse
Gpat1	Mm00833328_m1	Mouse
Lipe	Mm00495359_m1	Mouse
Lpin1	Mm00550511_m1	Mouse
Lpin2	Mm00522390_m1	Mouse
Mgll	Mm0049274_m1	Mouse
Mlycd	Mm01245665_m1	Mouse
Noct	Mm00802276_m1	Mouse
Nr1d1	Mm00520708_m1	Mouse
Nr1h2	Mm00437265_g1	Mouse
Nr1h3	Mm00443451_m1	Mouse
Per2	Mm00478113_m1	Mouse
Plin2	Mm00475794_m1	Mouse
Plin5	Mm00508852_m1	Mouse
Pnpla2	Mm00503040_m1	Mouse
Pnpla3	Mm00504420_m1	Mouse
Ppara	Mm00440939_m1	Mouse
Scd1	Mm01197142_m1	Mouse
Srebf1	Mm00550338_m1	Mouse

Supplemental Figure 1

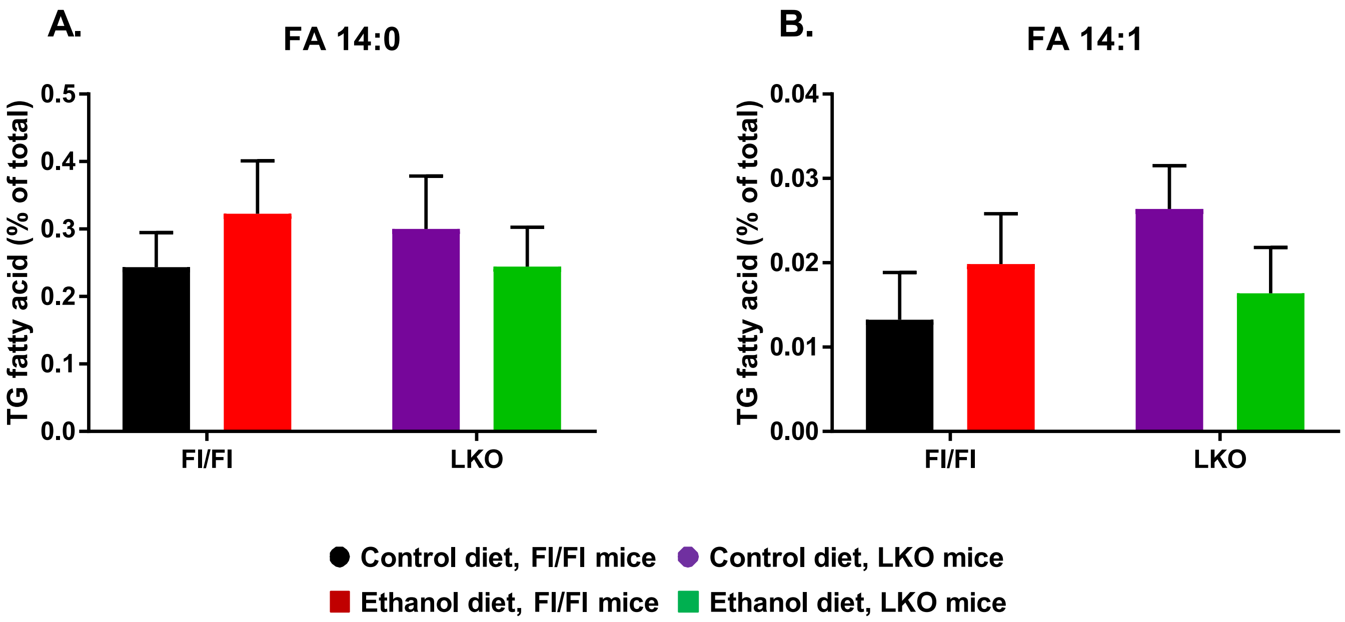

C.

Pairwise Comparison for TG FAs with Significant Genotype X Diet Interactions

Parameter	1 vs. 2	3 vs. 4	1 vs. 3	2 vs. 4
14:0	-	-	-	-
14:1	-	0.009	0.001	-

Results in black font are p-values ($p < 0.05$) for pair-wise comparisons between treatment groups. A dash indicates $p > 0.10$.

1 vs. 2 = Con Fl/Fl vs. EtOH Fl/Fl

3 vs. 4 = Con LKO vs. EtOH LKO

1 vs. 3 = Con Fl/Fl vs. Con LKO

2 vs. 4 = EtOH Fl/Fl vs. EtOH LKO

Supplemental Figure 1. Select hepatic triglyceride fatty acids altered by alcohol and liver clock disruption independent of time. Levels of fatty acids (FA) myristic acid (14:0; A) and myristoleic acid (14:1; B) as a percentage of the total triglyceride (TG) FA pool were determined using MS/MS^{ALL} as previously described in liver lipid extracts control-fed (black) and alcohol-fed (red) *Bmal1* Flox/Flox (Fl/Fl) and control-fed (purple) and alcohol-fed (green) *Bmal1* liver-specific knockout (LKO) mice at ZT 3 and ZT 15 (ZT 0: lights on/inactive period, ZT 12: lights offs/active period). Data are shown as mean $\pm$ SEM for n = 7-8 mice/genotype/diet with ZT 3 and ZT 15 averaged together. Results for pair-wise comparisons between groups are provided in (C).

Supplemental Figure 2

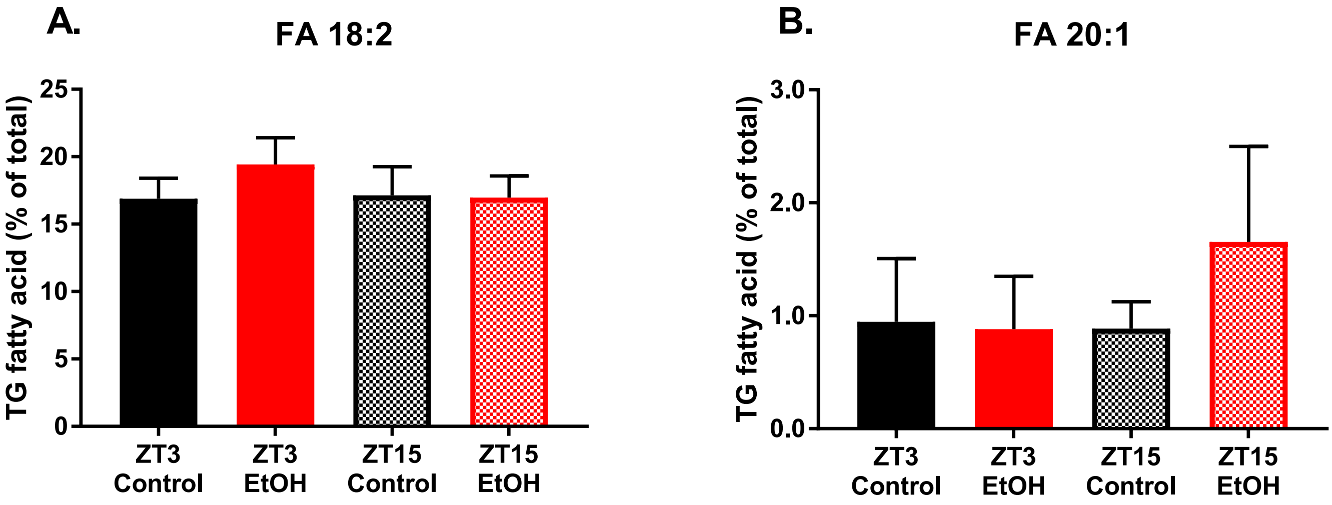

C.

Pairwise Comparisons for TG FAs with Significant Diet X Time Interaction

Parameter	1 vs. 2	3 vs. 4	1 vs. 3	2 vs. 4
18:2	0.01	-	-	0.02
20:1	-	2.1E-4	-	2.7E-4

Results in black font are p-values ($p < 0.05$) for pair-wise comparisons between treatment groups. A dash indicates $p > 0.10$.

1 vs. 2 = Con ZT 3 vs EtOH ZT 3

3 vs. 4 = Con ZT 15 vs. EtOH ZT 15

1 vs. 3 = Con ZT 3 vs. Con ZT 15

2 vs. 4 = EtOH ZT 3 vs. EtOH ZT 15

Supplemental Figure 2. Time of day-dependent alterations in select hepatic triglyceride fatty acids independent of genotype. Levels of fatty acids (FA) linoleic acid (18:2; A) and eicosanoic acid (20:1; B) as a percentage of the total liver triglyceride (TG) FA pool were determined as previously described using MS/MS^{ALL}. Data were averaged across genotypes for control-fed mice (solid black) and alcohol-fed mice (solid red) at ZT 3 and control-fed mice (open black) and alcohol-fed mice (open red) at ZT 15 (ZT 0: lights on/inactive period, ZT 12: lights offs/active period). Data are shown as mean $\pm$ SEM for n = 7-8 mice/diet/time-point. Results for pair-wise comparisons between groups are provided in (C).
